# Preliminary report of a simulation community of practice needs analysis

**DOI:** 10.1186/s41077-020-00130-4

**Published:** 2020-07-01

**Authors:** Monica Peddle, Karen Livesay, Stuart Marshall

**Affiliations:** 1 School of Nursing and Midwifery, College of Science, Health and Engineering, La Trobe University, Kingsbury Drive, Bundoora, 3086 Australia; 2 School of Health and Biomedical Sciences, College of Science Engineering and Health, RMIT University, Melbourne, Australia; 3 Anaesthesia Teaching & Research, Monash University, Melbourne, Australia

**Keywords:** Health simulation, Pedagogy, Simulation training, Needs analysis

## Abstract

**Aim:**

To understand the current needs related to education and training, and other investment priorities, in simulated learning environments in Australia following a significant period of government funding for simulation-based learning.

**Methods:**

A mixed methods study, comprising qualitative focus groups and individual interviews, followed by a quantitative cross-sectional survey informed by themes emerging from the qualitative data.

**Findings:**

Two focus groups and 22 individual interviews were conducted. Participants included simulation educators, technical users and new adopters. Survey data were collected from 152 responses. Barriers at the introduction and maintenance stages of simulated learning included irregular staff training resulting in inconsistent practice, and lack of onsite technical support. Educators lacked skills in some simulation and debriefing techniques, and basic education and research skills were limited, while technicians raised concerns regarding the maintenance of equipment and managing budgets.

**Discussion and conclusion:**

Despite its effectiveness as an education tool, barriers remain at the introduction and maintenance stages of simulated learning environments. Efforts to improve the integrity and sustainability of simulation training should be informed by a comprehensive needs analysis. The resulting data should be used to address barriers in a way that maximises the limited resources and funding available for this important learning tool.

## Introduction

Simulation-based education is known to be costly to set up, implement and maintain [1]. However, benefits from this education strategy such as developing knowledge and skills, while improving critical thinking and confidence [1–3], suggest that investment in simulation-based learning should lead to enhanced patient outcomes. It is necessary to understand the gaps occurring at both inception of new simulation centres and services as well as for operating simulated learning environments, to ensure integrity and sustainability of this important education tool [4, 5].

Significant funding was released in 2012 to facilitate the establishment of and improve the capacity for simulation-based learning in Australia. A total of $94 million was invested by the federal government to fund projects around clinical training reform within simulated learning environments. The purpose of undertaking this study was to evaluate the current state of simulated learning environments in Victoria, Australia, post-government investment into simulation-based learning for health professionals to improve training capacity. The study sought to ascertain gaps in knowledge, skills and behaviours, or resources and infrastructure to identify further investment priorities and was commissioned by the state government. While this study focused on a distinct geographical region in Australia, the findings give rise to transferable concepts that enable a broader audience to make connections with our findings and their own contexts. Additionally, this paper provides a clear approach to conducting a needs analysis for others to replicate.

### Background

While many countries have had significant investment in developing simulation programmes to support patient safety, reduce medical errors, develop practitioner competency and confidence, and to build safe, quality healthcare systems [6], there is limited reporting of the evaluation of the investment or requirements for ongoing resourcing of simulated learning environments post-initial investment [1]. It is important for post-investment needs analysis to be conducted, to evaluate if funding has achieved programme aims and to direct further resources in the most appropriate and effective manner. Moreover, it is likely that other impediments and challenges will have arisen post-funding that may impact on, delay or deter the implementation and sustainability of quality simulation activities. Studies such as this, while contextual, may influence or inform further investigation and reporting of strategies and resources required to maintain simulation programmes. Reporting findings of such needs analysis would enable sharing of information to inform future funding regarding simulation-based education.

The overall aim of undertaking this study was to identify investment priorities for improving knowledge, skills and behaviours in simulation, or simulation resources and infrastructure in the state of Victoria, Australia. A comprehensive needs analysis was guided by the question: What are the current needs of the Victorian simulation community related to education and training, and other investment priorities? This paper reports part of a larger study and focuses on priorities for improving knowledge, skills and behaviours of simulation personnel across the simulation community of practice in Victoria.

## Method

### Study design

**Fig. 1:**
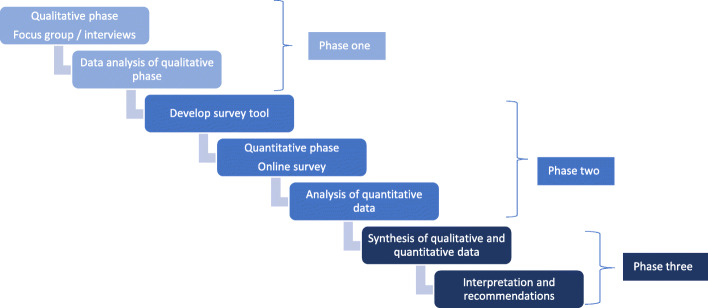
Exploratory sequential study design

We used a sequential exploratory mixed methods approach, comprising qualitative focus groups and individual interviews, followed by a quantitative cross-sectional survey informed by themes recognised in the qualitative data (Fig. 1). Sequential exploratory mixed methods were deemed appropriate for this study as the qualitative phase was required to identify study variables and develop suppositions, and inform development of a targeted and current, quantitative survey tool [7]. In the quantitative phase, answers will be explored in terms of the correlation of importance and confidence within each of the stakeholder groups. Data were collected in 2017. The study was guided by a project advisory group comprised of experts in health simulation from various stakeholder groups including medicine, nursing, allied health, government, higher education, continuing professional development and professional simulation associations. Metropolitan and regional areas were represented.

### Ethics

Ethical approval to conduct the project was granted by the La Trobe University Human Ethics Committee (ethics reference number S2000000322). Participants received an overview of the objectives, scope and purpose of the study. Written consent was obtained prior to focus groups and individual interviews. Implied consent was indicated by the completion of the online cross-sectional survey. Data were deidentified to maintain confidentiality.

### Population and sampling

A purposive sampling approach was used in the qualitative phase to identify suitable participants to address the research question. The authors sought representatives of simulation personnel across different population groups including professions, organisations, simulation modalities, levels of faculty experience, role in simulation and geographical locations. Nominations for representatives were sought from the advisory group and industry partners, with the advisory group making the final recommendations. Invitations to participate in interviews or focus groups were emailed to 46 participants representing each of the designated groups. Two focus groups (*n* = 8) and 22 individual interviews were completed with a total of 30 participants (*n* = 30). Participants self-assigned to a focus group or because of limited availability were interviewed individually. Focus groups enabled the identification of similarities and differences between user groups, while interviews supported gaining in-depth information.

In the quantitative phase, participants were recruited by ‘snowball sampling’ and word of mouth. Invitations to participate were emailed to contacts of the advisory group and self-identified communities of practice, associations or professional groups with anticipated simulation users with a request to forward the invitation amongst their network and members (Table 1). Participants who participated in the qualitative phase were invited to participate in the quantitative phase. Finally, links to the survey were circulated using multiple social media platforms to achieve broader dissemination. The survey remained open for 3 weeks. A total of 107 usable responses were collected.

**Table 1:** The specific communities of practice, associations and groups who disseminated the invitation amongst their members

College, society, association or group	
Victorian Simulation Alliance	
Royal College of Surgeons	
Australians Nurse Teachers Society	
Australasian College of Emergency Medicine	
Victorian Council of Social Service - Industry Training Advisory Board	
College of Oral Health Academics	
Victorian Department of Health and Human Services (DHHS) Simulation Expert Contact database	

### Phase 1—data collection and analysis

The first phase of data collection comprised a qualitative exploration that would inform the later, detailed work. The purpose of this stage was to collect ideas, concerns and predictions, as well as identify strengths and limitations related to simulation-based education within the simulation community of practice in the Australian state of Victoria. The expert project advisory group endorsed a schedule of open-ended questions (Supplementary file 1), designed to illicit participant perspectives and encourage participants to take a ‘blue sky’ approach to their assessment of future needs in the field. Review of the question schedule by the advisory group enabled quality to be maintained in the study and limit the influence of researcher values and beliefs on questions asked. Interviews and focus groups were conducted with participants at mutually convenient times over the phone or face-to-face. Focus groups and individual interviews were audio-recorded and transcribed for analysis.

Thematic analysis was used to identify themes and elements in all qualitative data. In phase 1, two researchers independently coded each transcript in the qualitative data analysis software (QSR NVivo, version 10). The final themes and elements were decided on consensus from researchers.

### Phase 2—data collection and analysis

The second phase of data collection was a quantitative cross-sectional survey. Themes and elements from the qualitative phase informed the development of items in a cross-sectional survey administered via Qualtrics^xm^ (Qualtrics LLC, Sydney, Australia). Previous needs analysis surveys conducted by the funding organisation were reviewed, and items scrutinised for consistency, coherence and fit with themes and elements identified through phase 1. Items that displayed confluence according to the researchers were added to the survey. New items were developed and added to the survey upon consensus from researchers. Items were grouped into branching decision trees to meet specific requirements of different user groups and contained open-ended, Likert scale responses and ordinal scale items. The expert project advisory group reviewed the developed cross-sectional survey to ensure the items were robust, current, applicable and well designed. Based on feedback, minor adjustments were made to question construction and survey structure for clarity. The final survey tool was piloted with 10 participants from each of the representative groups equally distributed over metropolitan and regional locations. Minor changes to response options and question items were made to refine usability, clarity and face and content validity. The final survey tool comprised 5 sections and 47 items (Supplementary file 1). Questions relating to confidence and importance of a topic used similar Likert response scores allowing a direct comparison to be made. The premise of using these measurements is that confidence and importance are correlated with each other (Giezendanner et al., 2017). If an item had high importance and a low confidence, a perceived skill or knowledge gap was assumed to exist.

Descriptive statistics and nonparametric pairwise comparisons were used to analyse quantitative data using IBM® SPSS® Statistics (version 20.0). For the descriptive statistics, the *n*, mean, std. deviation, median, minimum, maximum, range, skewness, kurtosis, and std. error of mean for each item were calculated (Supplementary file 1). For the correlation between different items in a multi-item scale, we have used the nonparametric ‘Spearman rank-order correlation coefficient’, known as ‘Spearman’s rho’ or ‘Spearman’s *ρ*’. For the test of normality, we have used the Shapiro-Wilk test. For comparison of two matched samples (i.e. a paired difference test), we have used the nonparametric ‘Wilcoxon signed-rank test’. Internal consistency when considering a pair of confidence and importance scales was measured using Cronbach’s alpha. In phase 2, open-ended responses in the cross-sectional survey were independently analysed by one researcher, with illustrative quotes selected to illuminate findings.

### Phase 3

Phase 3 involved the synthesis of qualitative and quantitative results using triangulation. Triangulation enables a comprehensive view of the data through cross-verification between the component elements, in this case, the qualitative and quantitative phases. Preliminary findings were presented to the expert project advisory group for discussion, with final recommendations developed via consensus from the advisory group and researchers.

## Results

### Phase 1: interviews and focus groups

A total of 30 participants participated in 2 focus groups (*n* = 8) and individual interviews (*n* = 22). Focus groups ranged from 48 to 55 min in duration with individual interviews ranging from 18 to 42 min in duration. Focus groups were conducted face-to-face (*n* = 1) and via video conference (*n* = 1). Interviews were conducted over the phone (*n* = 20) or via video conference (*n* = 2). Analysis of qualitative data identified 10 themes, each with multiple elements (Table 2).

**Table 2:** Themes and elements identified in phase 1 exploratory interviews and focus groups

Theme	Elements
Simulation	Implementing a broad range of simulation practices following best practice Simulated patient’s quality questioned with practice uninformed by theory Development of alternate modalities of simulation
Collaboration	Variability in collaboration and sharing resources Lack of collaboration between different stakeholders, hospital specialties and departments and hospital simulation centres Develop networking opportunities through communities of practice Regular networking between government, educators, trainers and hospitals (e.g. conferences, meetings, communication networks)
Recognition and incentives	Simulation not recognised as adding value to support backfill of staff off the ward and conducive rostering Infrastructure, leadership and executive support required to grow simulation programmes Promote opportunities of simulation to policymakers, educators, students and clinicians Promote certification, accreditation, benchmarking and minimum standards to enable recognition of staff and the work being done Professionalise education and training related to simulated learning environments to enable recognition of the specialised skill set
Teaching and learning	Evolvement of the educational pedagogy surrounding simulation Clinical skill development and assessment, and critical incident training well addressed but difficulties encountered in more complex areas such as behavioural and cultural change and interprofessional training Lack of understanding regarding surrounding design for learning to maximise learning from simulation experience Lack of standardisation in simulations used for assessments
Sustainability	Inadequate or unreliable funding sources Inequitable distribution of resources and spaces /funding across metropolitan and rural areas Purchase of equipment without conducting appropriate needs analysis Maintenance of equipment World-class simulation resources that need coordination and support Staff roles in simulation do not have definitive boundaries, many have multiple roles Lack of dedicated simulation staff including technical support Large turnover of simulation staff and excessive workloads
Professional development	Centralised training developed clarity in different roles in simulation Using a train-the-trainer model to build capacity in simulation in hospitals, across different disciplines Strategies to prevent skill decay and ongoing support and mentorship for simulation staff Availability of mentorship, education and training in regional areas Education and training for specific simulation modalities including in situ, simulated patients, high technology, immersive simulations, scenario design, hybrid simulation
Research	Need to improve research, publication and dissemination of activities and innovations from simulation in different settings Need to understand research priorities related to simulation and champion the outcomes achieved from simulation Form research collaboratives to develop research skills in clinical staff Sharing research being conducted in centres, organisations and groups
Patient safety	Supports strong operational teams: experience and initiative Using simulation to assess job design and workforce safety issues and usability testing of systems and equipment Apply simulation from a risk and harm minimization perspective Collaborate with quality and safety departments to manage risk
Technical	Clarify roles of simulation technicians Have dedicated technical support, so that educators can focus on training and not fixing equipment Audiovisual equipment knowledge Pressures of running a simulation supported by AV at the same time More technical training for all staff Develop the recognised role of simulation specialist
New adopters	Lack of understanding and experience regarding simulation and what it means Reluctance of some clinical communities to use simulation Lack of skills of staff in some work contexts to design scenarios for assessment

During the analysis of the qualitative data, the authors recognised three groups of stakeholders including simulation users, new adopters and technically focused users. Simulation users were described as competent or proficient in coordinating, facilitating, managing or assisting with simulation-based education. New adopters were novices who represented a discipline, sub-specialty or new work area that had not previously been included in health professional simulation education networks but were using simulation. Examples included social welfare staff, staff working in aged and community care, indigenous health, refugee health and residential care workers. Technically focused users were conceptualised as those people building scenarios or managing simulation facilities and equipment and programming manikins.

### Phase 2: cross-sectional survey

#### Demographic data

One hundred and sixty-six survey responses were collected, with 107 complete surveys used for the analysis. The breakdown of stakeholder groups represented in data was simulation users, 65% (*n* = 70); new adopter, 32% (*n* = 34); and technically focused users, 23% (*n* = 25). Due to the use of snowball sampling, respondents were from most states and territories in Australia; however, the greatest numbers were from metropolitan Victoria 82% (*n* = 87). The highest professional groups were nursing 50% (*n* = 53), medical 16% (*n* = 17) and midwifery 6% (*n* = 6) with minimal representation from allied health 4% (*n* = 4) and new work areas including community services and social work 2% (*n* = 4). Respondents worked predominantly in acute care 55% (*n* = 59) and higher education 49% (*n* = 52). Of the respondents, 62% (*n* = 66) had completed basic simulation training or a higher degree 46% (*n* = 49); however, 19% (*n* = 20) had no qualifications in simulation.

**Fig. 2:**
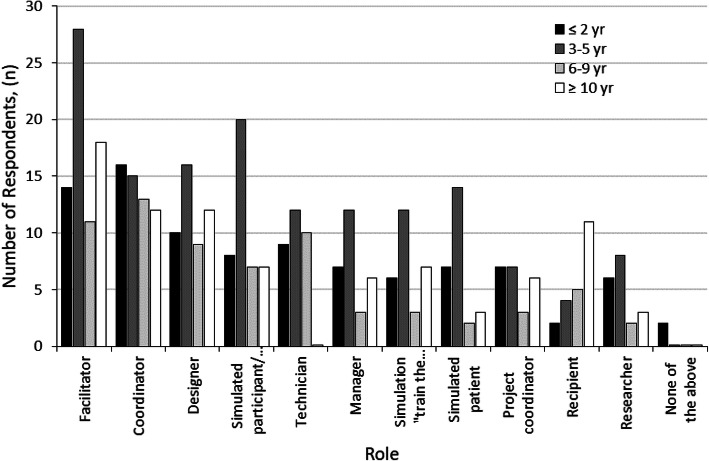
Plot of the number of respondents resolved by both role and experience

Staff in teaching roles had a median of 6 years of experience, compared with medians of 5 years for technical staff and 3 years for researchers. Predominant simulation audiences were undergraduates (*n* = 75) and continuing profession development (*n* = 69). New graduates (*n* = 55) and postgraduate specialist (*n* = 47) learners made up the rest of the reported learner groups. Data identified that participants fulfilled several roles in simulation with operational aspects including teaching, coordinating and designing simulation most common (Fig. 2). Being a participant or actor in simulation activities was a common role for participants with the second highest number of years in experience (Fig. 2). Notably, research and being a recipient of simulation were not common (Fig. 2). As respondents could choose multiple roles, the total responses are counted (Fig. 2).

#### Summary of findings—new adopters

**Fig. 3:**
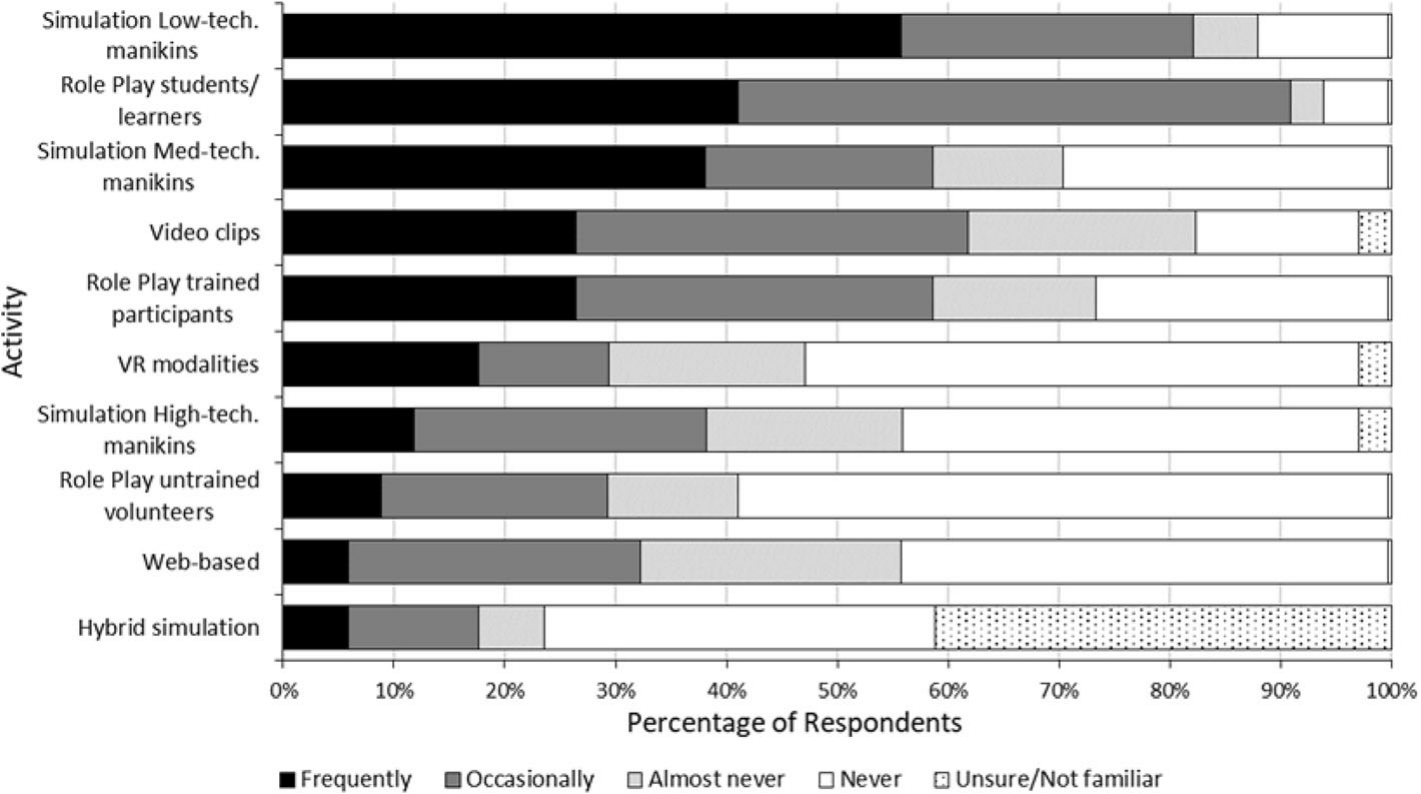
Plot of respondent percentage data related to simulation activities used in the last 12 months ranked from top to bottom according to decreasing frequency of use

**Fig. 4:**
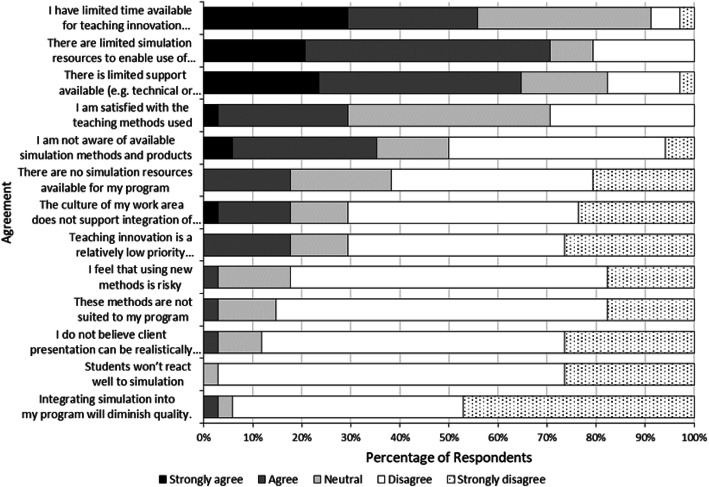
Plot of respondent percentage data ranked from top to bottom according to decreasing significance of barriers to integration of simulation in programme

Findings indicated that new adopters were familiar with and utilised simulation approaches using low technology modalities, including role plays and low technology manikins, while less common modalities of simulation aligned with higher forms of technology including web-based, virtual reality and high technology manikins (Fig. 3). The least common areas where new adopters use simulation, managing challenging situations (*n* = 7, 23%), correlated with the area that most new adopters indicated they would like to use simulation (*n* = 23, 77%). Respondents indicated they currently use simulation to standardise experience for learners (*n* = 11 28%) and to provide realistic workplace experiences (*n* = 11, 28%). Future applications would be aimed at addressing issues raised by placement providers (*n* = 24 89%) and supporting placement supervisors (*n* = 23, 58%). The barriers identified by respondents for the integration of simulation-based learning were related to limited time (*n* = 29 61%), resources (*n* = 24, 62%) and support (*n* = 22, 52%) (Fig. 4).

#### Summary of findings—technically focused users

Respondents indicated being identified as a ‘technician’ posed tensions, as individuals were not always employed or classified as technicians and the roles undertaken by this group did not always align with technical activities. Daily or weekly roles undertaken by this group were adopting a support role in scenarios, working directly with learners, orientating learners and developing scenarios with faculty. Less common roles were building programmes using the simulator software and participating in research. All technical users surveyed were confident in all aspects of preparing simulation environments.

**Fig. 5:**
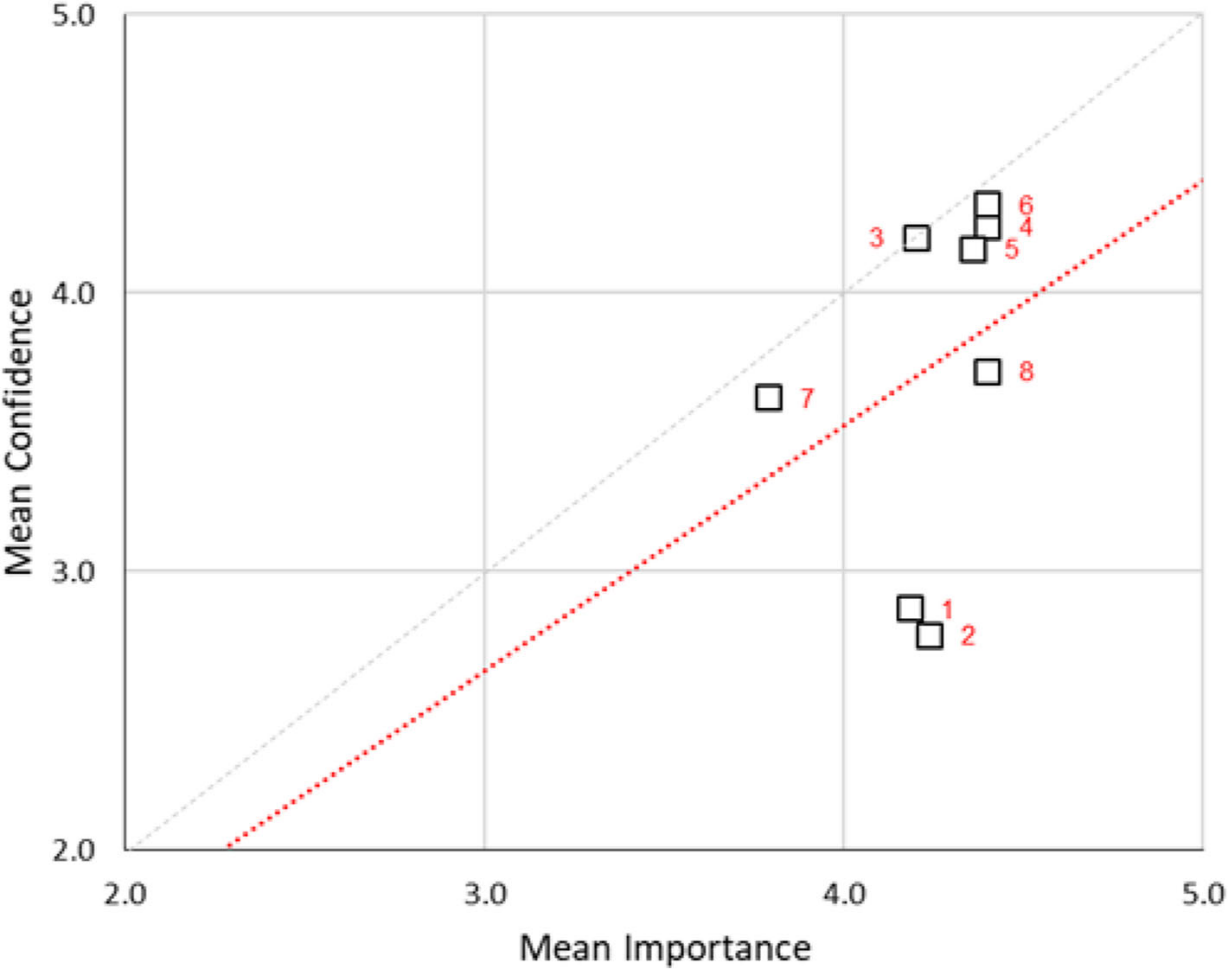
Scatterplot of mean confidence vs. mean importance technician’s knowledge. The red dotted line is the OLS (ordinary least-squares) line of best fit to the data (in the form *y* = m*x*). The grey dotted line is the 1:1 line (i.e. where mean confidence = mean importance)

Correlations between importance and confidence ranked on a 10-point scale were statistically analysed using Spearman’s rho with a modest degree of overlap in confidence scale (*ρ* = 0.21) and a low degree of overlap in the importance scale (*ρ* = 0.14). The relationship between the means of confidence and importance scale is displayed in a scatter plot (Fig. 5). While scale items for healthcare terminology (3), laptops and simulator software (4), manikin technology (5), medical equipment and consumables (6) and moulage (7) lie fairly close to the 1:1 line, indicating a fairly good matching of assigned confidence to assigned importance, scale items related to developing budgets (1), developing business cases (2) and operating AV equipment (8), lie considerably below the 1:1 line, indicating that for these items assigned importance considerably outstrips assigned confidence. Results of the (Shapiro-Wilk) test for normality on the aggregated mean responses indicate the data departs significantly (*p* < 0.05) from normality. This indicates that a nonparametric test for differences between the matched mean responses should be used (e.g. the Wilcoxon signed-rank test). Results of the Wilcoxon signed-rank test on the matched differences between aggregated mean responses indicate responses are different to a significant degree, at the *p* < 0.01 level. The values for Cronbach’s *α* are ~ 0.64 in both cases, which while low would be deemed satisfactory for a preliminary research investigation.

**Fig. 6:**
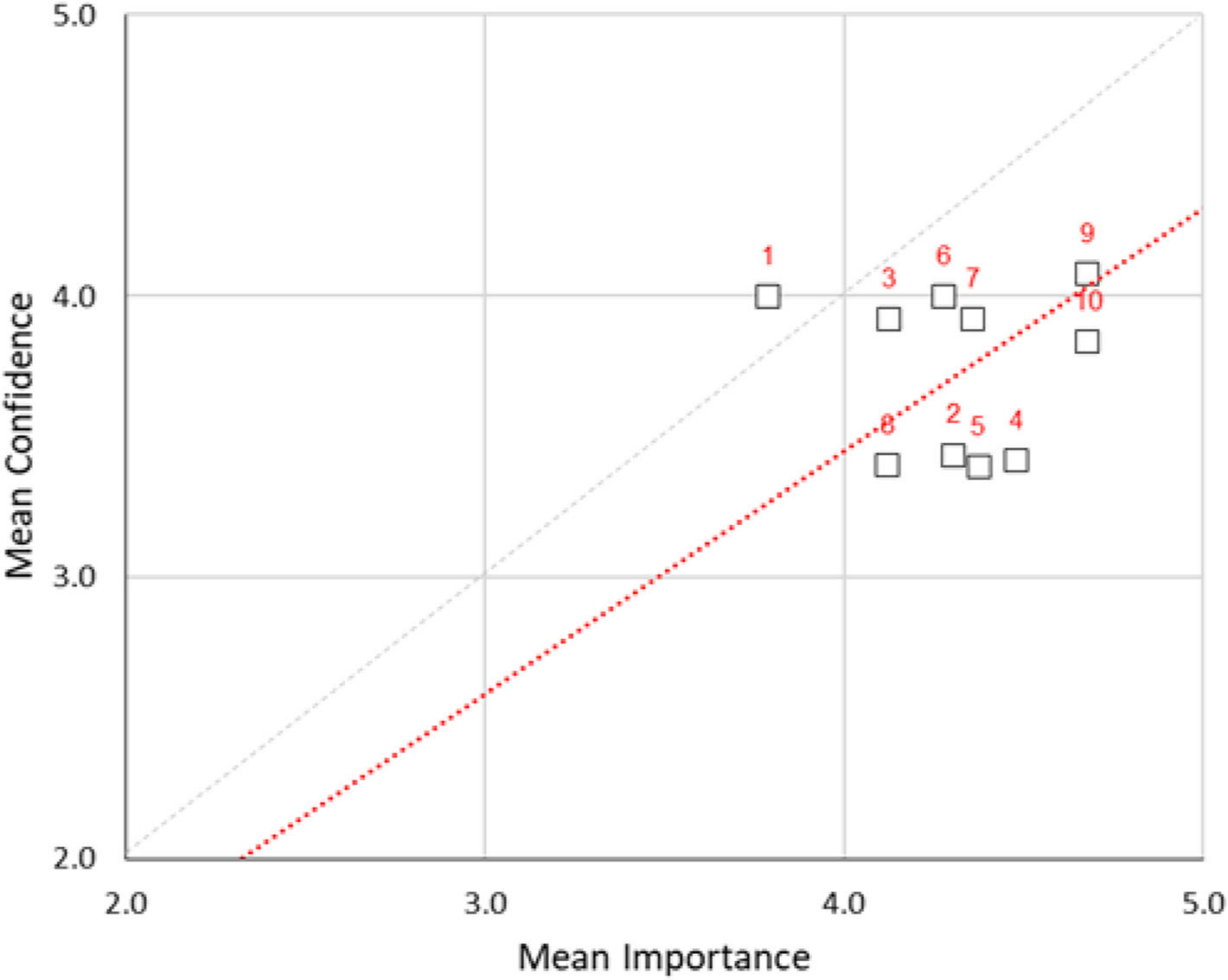
Scatterplot of mean confidence vs. mean importance in technicians’ skills

The correlation between confidence and the importance of specific skills for technicians returned a correlation coefficient indicating a moderate degree of overlap between scale items in confidence (*ρ* = 0.50) and importance (*ρ* = 0.43). In the scatter plot, nearly all of the scale items lie below the 1:1 line; this is especially so for items related to developing and implementing processes for equipment replacement (2), developing and maintaining schedules for maintenance (4), managing asset registers including repairs maintenance loans, etc. (5). Programming different simulator makes and models (8) and troubleshooting equipment failure (10) (Fig. 6). This indicates that for half of the scale items, assigned importance rather outstrips assigned confidence. The data departs significantly (*p* < 0.05) from normality with the responses different to a significant degree, at the *p* < 0.01 level. The values for Cronbach’s *α* are ~ 0.91 in both cases. This is considered a quite good reliability result, indicating that this scale may represent a useful instrument for what it proposes to measure.

#### Summary simulation users

**Fig. 7:**
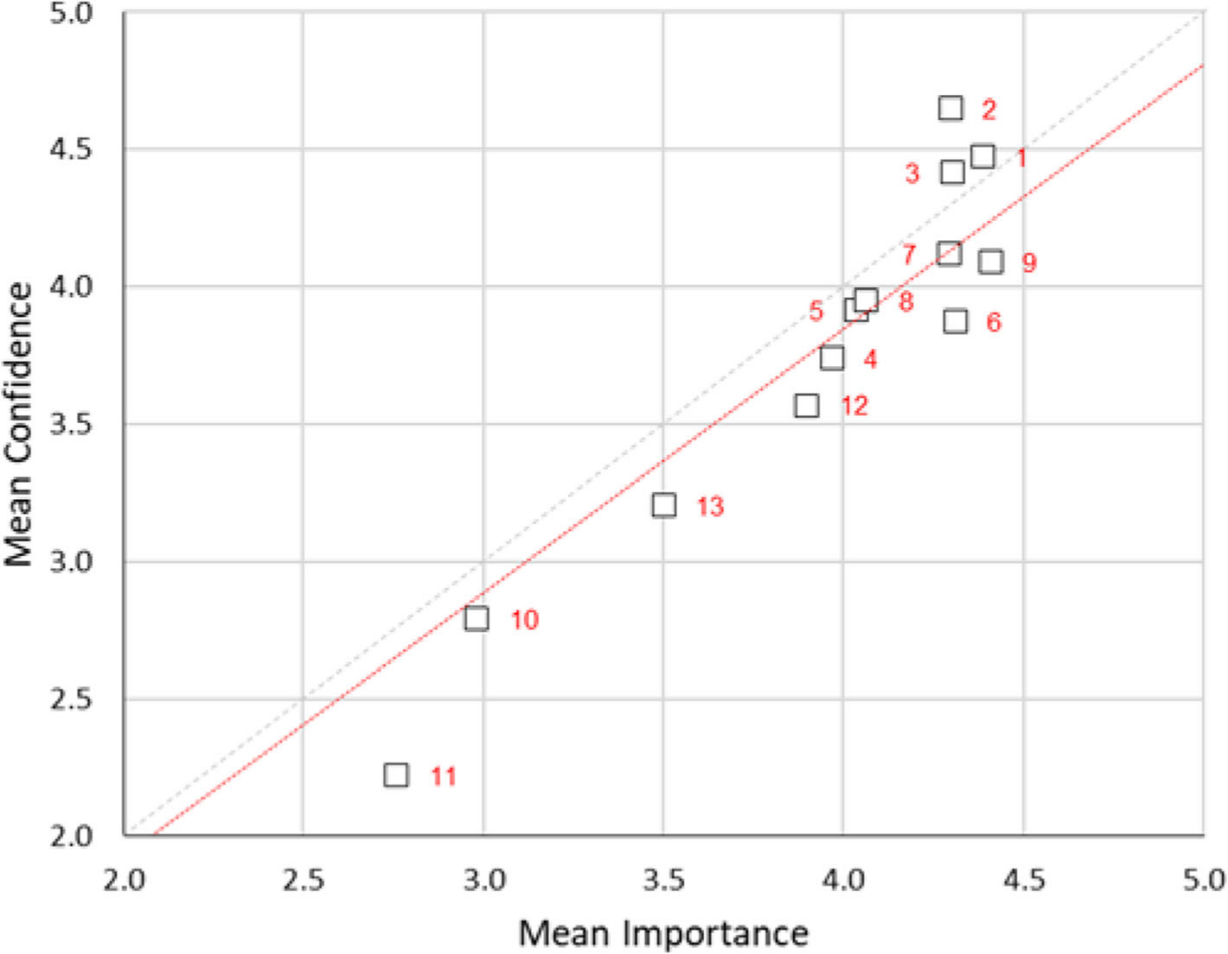
Scatterplot of mean confidence vs. mean importance for simulation users in simulation modalities

Correlations between confidence and importance of specific simulation modalities by simulation users identified a modest degree of overlap (*ρ* = 0.26). In the scatter plot, there appears a systematic mismatch between assigned confidence and assigned importance across the 13-scale items. For high confidence items including part task trainer (1), low technology manikins (2) and medium technology manikins (3) predominate with confidence higher than importance. At the lower end of the importance and relatively low confidence virtual reality-based simulation (11), observational simulation techniques (12) and time-sequenced simulations (13) predominate. The data does not depart from normality to a significant degree (at the *p* < 0.05 level). This indicates that the simple paired-sample *t* test for the difference between the distributions was appropriate. However, for consistency, the nonparametric Wilcoxon signed-rank test was used in all cases. The Wilcoxon signed-rank test confirms that the difference between assigned confidence and assigned importance, overall, is significant at the *p* < 0.05 level. The values for Cronbach’s *α* are in the range of 0.80-0.85. This is considered a good reliability result, indicating that this scale may represent a useful instrument for what it proposes to measure (Fig. 7).

**Fig. 8:**
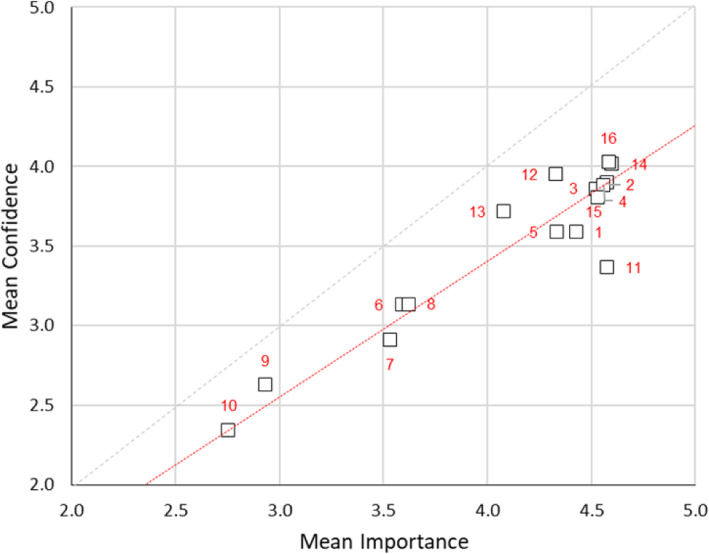
Scatterplot of mean confidence vs. mean importance for simulation users in work items

Spearman’s *ρ* indicates a moderate degree of overlap between the scale items in the simulation user’s education and training elements for confidence (*ρ* = 0.50) and importance (*ρ* = 0.36). The scatter plot indicates all 16 scale items including conducting learning needs analysis (1), integrating simulation activities into curricula (2), constructively aligning scenarios with ILOs (3), planning/conduct evaluation activities (4), design scenarios to address quality/risk data (5), simulated patient recruitment (6), simulated patient training programme (7), moulage techniques (8), computer or web-based simulation (9), virtual reality-based simulation (10), sustainability of simulation programmes (11), formative (12) and summative (13) assessment, teaching non-technical skills (14), interprofessional education (15) and team-based training (16) lie consistently below the 1:1 line. The data departs significantly (*p* < 0.01) from normality with the Wilcoxon signed-rank test on the matched differences different to a significant degree, at the *p* < 0.01 level (Fig. 8).

#### Summary of findings—professional development

**Fig. 9:**
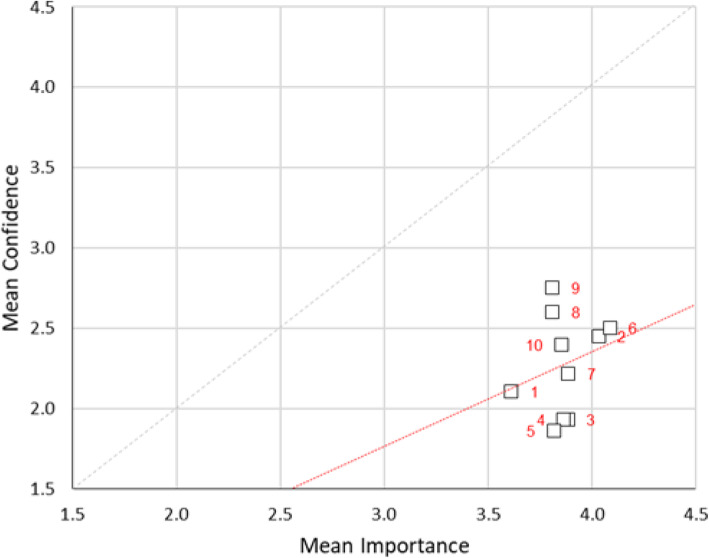
Scatterplot of mean confidence vs. mean importance for the ten items in research related to simulation in their profession/work area

The correlation between importance and confidence in the elements of research returned a high degree of correlation between the importance *ρ* = 0.65 and confidence *ρ* = 0.63. All 10-scale items including developing research protocols (1) and developing and using tools and instruments to measure outcomes (2), locating (3), applying for (4) and developing budgets for grant funding (5), writing and reporting project outcomes (6) including conference abstracts (8), conference presentations (9) and for writing papers for publication (7) as well as research ethics (10) lie far below the 1:1 line. In this case, the assigned importance far outstrips the assigned confidence, overall. The data departs significantly (*p* < 0.01) from normality with the Wilcoxon signed-rank test on the matched differences different to a significant degree, at the *p* < 0.01 level. The values for Cronbach’s *α* are ~ 0.95 in both cases representing good reliability, indicating that this scale may represent a useful instrument for what it proposes to measure (Fig. 9).

## Discussion

This study indicated several perceived gaps in knowledge, skills and behaviours of user groups identified in this research. These gaps pertain to the areas of ongoing training and education, research and application of simulation to areas other than education. There was confluence between the qualitative and quantitative findings, although because of the nature of the survey, some themes were explored in more significant detail in the interviews and focus groups.

Findings of this study identified three user groups including simulation users, new adopters and technically focused users. However, it was clear from the findings that there are no definitive boundaries between user groups, as many participants fulfilled several roles in simulation including facilitating, coordinating and managing as well as being a participant or actor in simulations and running audio-visual equipment. As evidenced by the quote:

We’re having to be our own technicians, we’re having to be our own educational experts, we have to be able to debrief, we have to do it all, and in fact it’s actually really hard to do all that well, or at all, without the appropriate support. (FG-P1)

Similarly, Crawford, Monks, Bailey, and Fernandez [8] identify the multiplicity required in simulation staff roles. Therefore, education and training programmes need to consider the diversity present in roles related to simulation and cater for the specific membership of the group. Additionally, position descriptions need to be flexible to be able to be modified for different contexts and requirements an organisation may have.

The demographic data specifies that most participants have 3-5 years of experience as a facilitator. Interestingly, the second highest years of experience were related to being a simulated participant or actor in the simulation activities. These findings reinforce previous literature affirming the multiple roles of simulation faculty [8]; however, it also presents an interesting discovery regarding the relevance of this experience in participating in simulations to developing expertise as simulation faculty. This finding has not previously been identified in simulation literature. Further research is required to investigate if and how experience as a simulated participant or actor in the simulation activity develops faculty expertise.

### New users

As expected for new users, there were significant perceived gaps in knowledge and skill that appear to stem from limited time for innovation, a lack of resources and expert support to implement these approaches in education programmes which aligns with elements in the qualitative findings. There was a gap in areas focused on higher technology simulation, such as virtual reality and high technology manikins, and simulated patient methodology. Challenges arose due to the conflict caused by the lack of awareness of simulation and gaps in basic training and education, and mandated simulation-based assessments in some programmes, as evidenced by:

People employed in education, in RTOs [Registered Training Organisation] that do not have the skills to develop scenarios yet scenarios to be used as assessment or an accrediting process, they’re falling very short. They’re inadequate and not well-planned scenarios that are actually having adverse effects on the staff that are involved in them, the learners and they’re not outcomes focused and so they’re not actually looking at what was needing to be measured in the first place. (FG-P5)

Adopting technologically advanced teaching requires commitment and competence [9]. To support new adopters to engage with simulation, contextualised exemplars could be provided that demonstrate expert performance in quality simulation scenarios using various simulation approaches across the learning continuum.

### Technically focused users

There was substantial diversity amongst the technically focused user group and a standardised term such as ‘technician’ had little unification as evidenced by:

My feeling is that our title sim technician or sim technologist is not descriptive of what we do … We all have so many different various backgrounds and roles … (FG-P6)

Survey results indicated that over 50% of technically focused users adopted support roles and worked directly with students in simulation. Additionally, over 40% of technically focused users reported collaborating with faculty to develop scenarios and 30% orientated participants to the environment. Bailey et al. [10] similarly documented a wide variation in technical staff tasks. These findings are supported in the qualitative data.

[On] the technical side I often play the patient behind the mike, I’ll be the voice of the patient for simulations … working through the scenarios and working out what's working well and taking a part in the whole simulation scenario activity … If the educator … is new to simulation … I’ll just sort of try and take them through, tell them where some information is, guide them … (FG-P2)

For technically focused users, survey data revealed eight knowledge and skill gaps, with respondents identifying areas aligning with elements in the qualitative data such as audio-visual equipment knowledge and running simulator and audio-visual simultaneously in simulation activities, with other areas aligning to business cases, budget development, maintenance schedules and asset registers. However, all technical users surveyed were confident in all aspects of preparing simulation environments. These findings suggest that technically focused users would benefit from education and training related to business and management courses.

### Simulation users

While simulation users reported using and feeling confident with simulation approaches, when applying approaches to their work, 13 knowledge and skill gaps were identified as problematic by the participants. Additionally, simulation users reported gaps in basic education skills such as learning needs analyses, assessment and integrating simulation in curricula. Positively, these findings indicate fundamental training and education programmes are utilised with over 62% of respondents undertaking identified simulation training programmes (National Health Education and Training in Simulation (NHETSim) and the Australian Simulation Educator and Technician Training (AUSETT) Program). However, ongoing perceived gaps suggest existing training packages may not cater for or penetrate to new adopters from areas outside traditional healthcare, such as the emerging users from community services including disability support and youth workers. Additionally, as simulation-based education is only one component of learning and teaching, a sound knowledge of pedagogy, design for learning including assessments, is required [11]. Moreover, without ongoing learning, fundamental knowledge and skills related to simulation may not be reinforced, embedded or extended [12]. Therefore, future programmes should enable contextualisation to specific work areas and support the application of knowledge and skills in practice and consolidation of learning via mentorship.

### Research development

These study results indicate a perceived gap as identified by participants may exist in knowledge and skills pertaining to research in Victorian simulation communities of practice, as evidence by statistically significant findings and supported by a theme and elements in qualitative data.

I think research is a huge area for us that’s a gap. We’re very concerned operationally, but we’re not really spending time writing about what we’re doing and what we’re learning, and that’s the thing, that’s an important area. (I2)

As a result, simulation innovation and research being done in local communities of practice are not being disseminated to the wider simulation community. There are significant risks that the Victorian simulation community will fall behind in research, innovation and simulation quality. Suggestions to redress these barriers include developing partnerships across stakeholders to develop research collaboratives, and local opportunities for dissemination of research activities [13]. While research training programmes may be useful to develop novice researchers, the better impact may be felt from enticements to undertake graduate research study such as scholarships for higher degrees.

#### Strengths and limitations

Strengths of this research relate to data collection across a variety of stakeholder groups. Use of interviews and focus groups enabled detailed information to be obtained. Themes and elements were jointly constructed with supporting data examined for coherency, consistency and fit, and conflicts were resolved through consensus. The cross-sectional survey was piloted, with feedback enabling revision, to ensure question clarity and face and content validity. Additionally, the systematic nature of the research and the level of detail articulated present opportunities for the research to be replicated. It is important to note that the researchers knew many participants which may have influenced data obtained.

While findings present insight into the Victorian simulation communities of practice, caution must be exercised as findings may differ if other stakeholder groups were involved. However, the methods used in this research are transferable. Additionally, most respondents in the survey were Victorian based; the influence of widening the survey to other locations nationally may impact findings. The short time frame for collecting survey responses, 3 weeks, undoubtedly impacted on the response rate. The length and complexity of questions in the survey could be considered as a limitation; however, the comprehensive survey enabled a significant level of detail and granularity.

## Conclusion

This paper reported on a needs analysis conducted to identify priorities for improving knowledge, skills and behaviours of simulation personnel across one simulation community of practice. The outcomes of this study categorised stakeholders in simulation into three groups, simulation user, new adopters and technically focused users, and revealed a lack of homogeneity in roles undertaken by staff in simulation. The themes and results from the qualitative and quantitative phases of this study were highly confluent with statistically significant findings in elements of skills, modalities and knowledge across multiple stakeholder groups aligning with the themes in the qualitative data. The findings enabled an insight into the current status of simulation and simulation personnel as a result of government investment. Moreover, it identified that future investment could be targeted at current gaps in simulation research expertise that may impact knowledge dissemination and engagement.

Further, this needs analysis outlines the ongoing cyclical process of and importance in identifying, supporting, developing and evaluating investments in simulation-based education aimed to develop capacity in learning and teaching and research. Importantly, this study provides a detailed description of a needs analysis process to enable others to replicate the process.

## Supplementary information

**Additional file 1:.** Supplementary tables and figures.

## Data Availability

All data relevant to this study are included in this published article and its supplementary information files.

## Abbreviations

SPSS: Statistical Package for the Social Sciences
RTO: Registered training organisation
NHETSim: National Health Education and Training in Simulation
AUSETT: Australian Simulation Educator and Technician Training
