# Supplementary material for: Preliminary report of a simulation community of practice needs analysis
Source: Adv Simul (Lond). 2020 Jul 1;5:11. doi: 10.1186/s41077-020-00130-4 (PMC7329516; doi:10.1186/s41077-020-00130-4)
Supplement: Supplementary file 1 — Additional file 1:. Supplementary tables and figures. [file 41077_2020_130_MOESM1_ESM.docx]

***‘New Adopters’***

| **Q13- Activities you have used in the last 12 months:** | | | | | | | | | | |  | |  |  |  |  |  |
| --- | --- | --- | --- | --- | --- | --- | --- | --- | --- | --- | --- | --- | --- | --- | --- | --- | --- |
|  | Hybrid  simulation | Web-based | Role Play  untrained volunt. | Simulation High-tech.  manikins | VR modalities | Role Play trained  participants | Video clips | Simulation Med-tech.  manikins | Role Play students/ learners | Simulation Low-tech.  manikins | |  |  |  |  |  |  |
| Question Label | Q13_10 | Q13_2 | Q13_5 | Q13_9 | Q13_3 | Q13_6 | Q13_1 | Q13_8 | Q13_4 | Q13_7 | |  |  |  |  |  |  |
|  | Number of Respondents | | | | | | | | | | |  |  |  |  |  |  |
| Frequently | 2 | 2 | 3 | 4 | 6 | 9 | 9 | 13 | 14 | 19 | |  |  |  |  |  |  |
| Occasionally | 4 | 9 | 7 | 9 | 4 | 11 | 12 | 7 | 17 | 9 | |  |  |  |  |  |  |
| Almost never | 2 | 8 | 4 | 6 | 6 | 5 | 7 | 4 | 1 | 2 | |  |  |  |  |  |  |
| Never | 12 | 15 | 20 | 14 | 17 | 9 | 5 | 10 | 2 | 4 | |  |  |  |  |  |  |
| Unsure/Not familiar | 14 | 0 | 0 | 1 | 1 | 0 | 1 | 0 | 0 | 0 | |  |  |  |  |  |  |
| Total n | 34 | 34 | 34 | 34 | 34 | 34 | 34 | 34 | 34 | 34 | |  |  |  |  |  |  |
|  | Percentage of Respondents | | | | | | | | | | |  |  |  |  |  |  |
| Frequently | 5.9 | 5.9 | 8.8 | 11.8 | 17.6 | 26.5 | 26.5 | 38.2 | 41.2 | 55.9 | |  |  |  |  |  |  |
| Occasionally | 11.8 | 26.5 | 20.6 | 26.5 | 11.8 | 32.4 | 35.3 | 20.6 | 50.0 | 26.5 | |  |  |  |  |  |  |
| Almost never | 5.9 | 23.5 | 11.8 | 17.6 | 17.6 | 14.7 | 20.6 | 11.8 | 2.9 | 5.9 | |  |  |  |  |  |  |
| Never | 35.3 | 44.1 | 58.8 | 41.2 | 50.0 | 26.5 | 14.7 | 29.4 | 5.9 | 11.8 | |  |  |  |  |  |  |
| Unsure/Not familiar | 41.2 | 0.3 | 0.3 | 2.9 | 2.9 | 0.3 | 2.9 | 0.3 | 0.3 | 0.3 | |  |  |  |  |  |  |
| Total % | 100 | 100 | 100 | 100 | 100 | 100 | 100 | 100 | 100 | 100 | |  |  |  |  |  |  |

**Table C1.** **Number and percentage of respondents resolved according to the frequency with which they have engaged in certain simulation activities in the last 12 months. (Likert scale 1 – 5: Unsure/Not familiar – Frequently).**


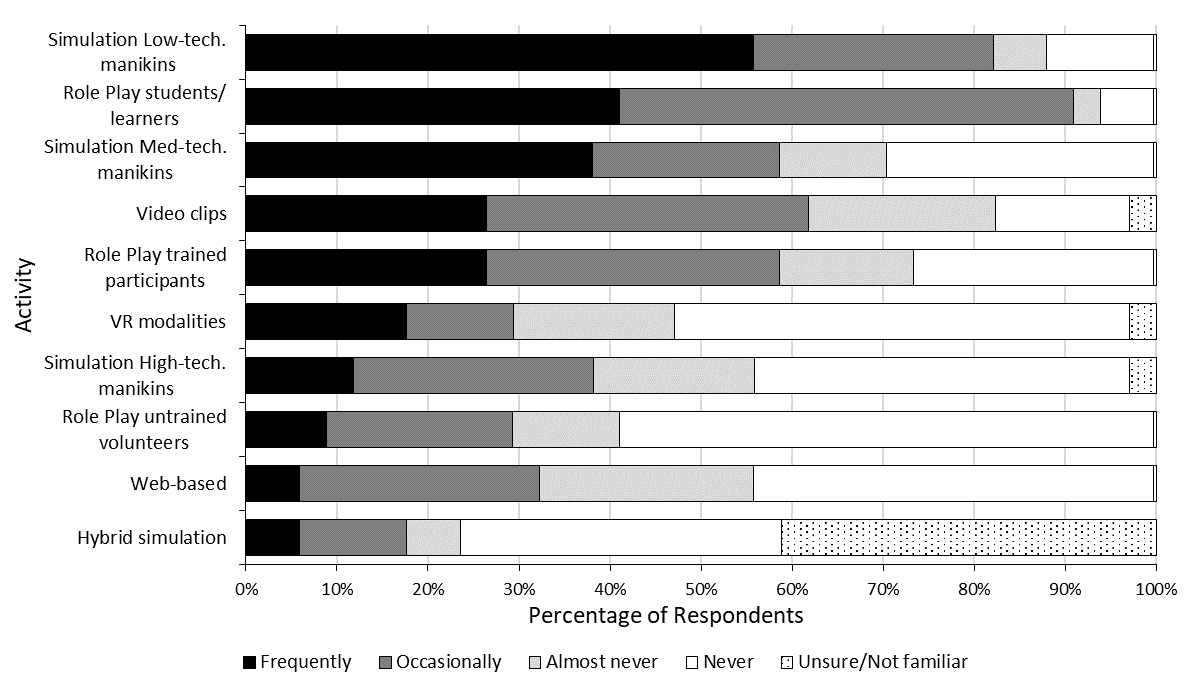


**Figure C1.** **Plot of respondent percentage data from Table C1 above. Ranked from top to bottom according to decreasing frequency of use.**

| **Q15 - Indicate if you are currently using simulation, or would like to use simulation in the following areas related to learning:** | | | | | | | | | | | |
| --- | --- | --- | --- | --- | --- | --- | --- | --- | --- | --- | --- |
|  | Managing challenging  behaviours | Developing  Reflective practice | Inter-professional  practice | Increasing  critical thinking | Instilling appropriate  professional behaviour | Improving clinical  reasoning skills | Support/anchor  the application of  theory and content | Integrating prior  knowledge/skills | Improving student  competence | Preparing students  for practice | Increasing student  confidence |
| Question Label | Q15_2 | Q15_8 | Q15_1 | Q15_3 | Q15_7 | Q15_10 | Q15_6 | Q15_4 | Q15_5 | Q15_11 | Q15_9 |
|  | Number of Respondents | | | | | | | | | | |
| Currently using | 7 | 15 | 15 | 16 | 16 | 19 | 20 | 24 | 24 | 23 | 24 |
| Would like to use | 23 | 19 | 17 | 18 | 17 | 13 | 12 | 9 | 9 | 8 | 8 |
| Not familiar | 0 | 0 | 0 | 0 | 0 | 0 | 1 | 1 | 0 | 0 | 0 |
| Total n | 30 | 34 | 32 | 34 | 33 | 32 | 33 | 34 | 33 | 31 | 32 |
|  | Percentage of Respondents | | | | | | | | | | |
| Currently using | 23.3 | 44.1 | 46.9 | 47.1 | 48.5 | 59.4 | 60.6 | 70.6 | 72.7 | 74.2 | 75.0 |
| Would like to use | 76.7 | 55.9 | 53.1 | 52.9 | 51.5 | 40.6 | 36.4 | 26.5 | 27.3 | 25.8 | 25.0 |
| Not familiar | 0.0 | 0.0 | 0.0 | 0.0 | 0.0 | 0.0 | 3.0 | 2.9 | 0.0 | 0.0 | 0.0 |
| Total % | 100 | 100 | 100 | 100 | 100 | 100 | 100 | 100 | 100 | 100 | 100 |

**Table D1. Number and percentage of respondents resolved according to their use, or desire to use, simulation in certain learning related areas.**


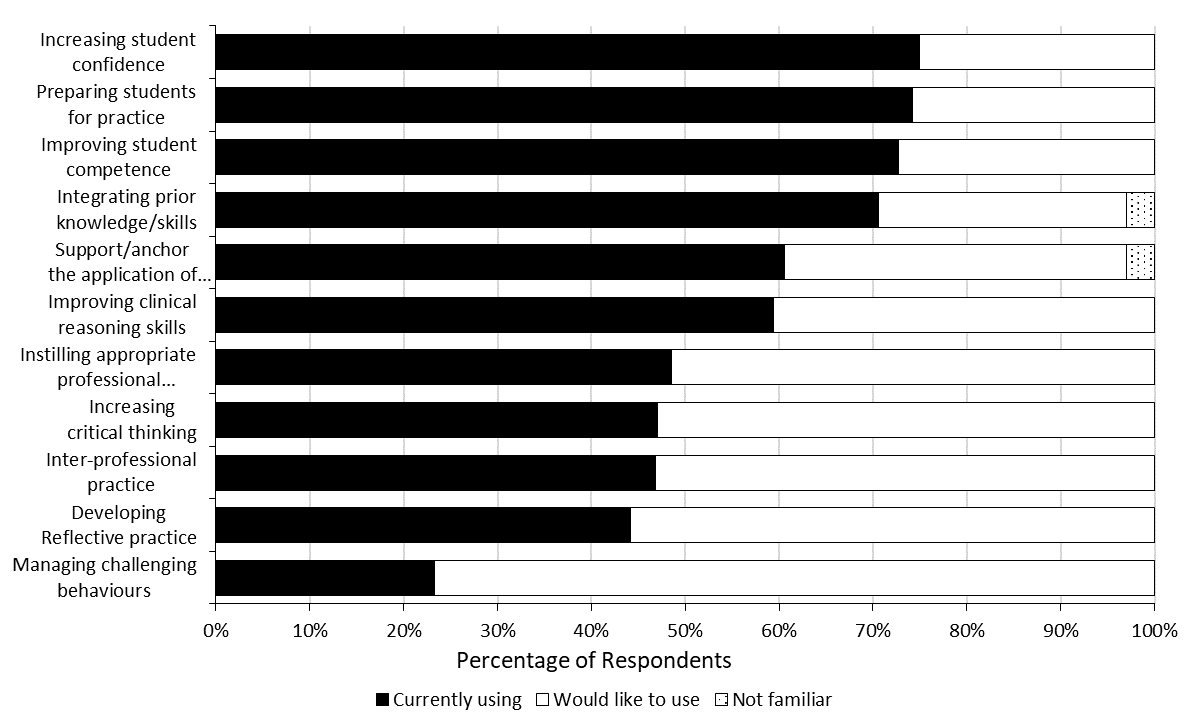


**Figure D1. Plot of respondent percentage data from Table D1 above. Ranked from top to bottom according to decreasing current use of activity (and therefore increasing wish to use activity).**

**Section E – Question 16
*[Answered only by those who self-identified as ‘New Adopters’ in Q12, n ≈ 34]***

| **Q16 - Indicate if you are currently using simulation, or would like to use simulation in the following areas related to assessment:** | | | |
| --- | --- | --- | --- |
|  | Improving inter-rater  reliability in assessment | Evaluating student  performance | Assessment  (of student) |
| Question Label | Q16_1 | Q16_2 | Q16_3 |
|  | Number of Respondents | | |
| Currently using | 5 | 16 | 16 |
| Would like to use | 24 | 15 | 15 |
| Total n | 29 | 31 | 31 |
|  | Percentage of Respondents | | |
| Currently using | 17.2 | 51.6 | 51.6 |
| Would like to use | 82.8 | 48.4 | 48.4 |
| Total % | 100 | 100 | 100 |

**Table E1. Number and percentage of respondents resolved according certain simulation activities related to assessment they are either currently using or would like to use.**


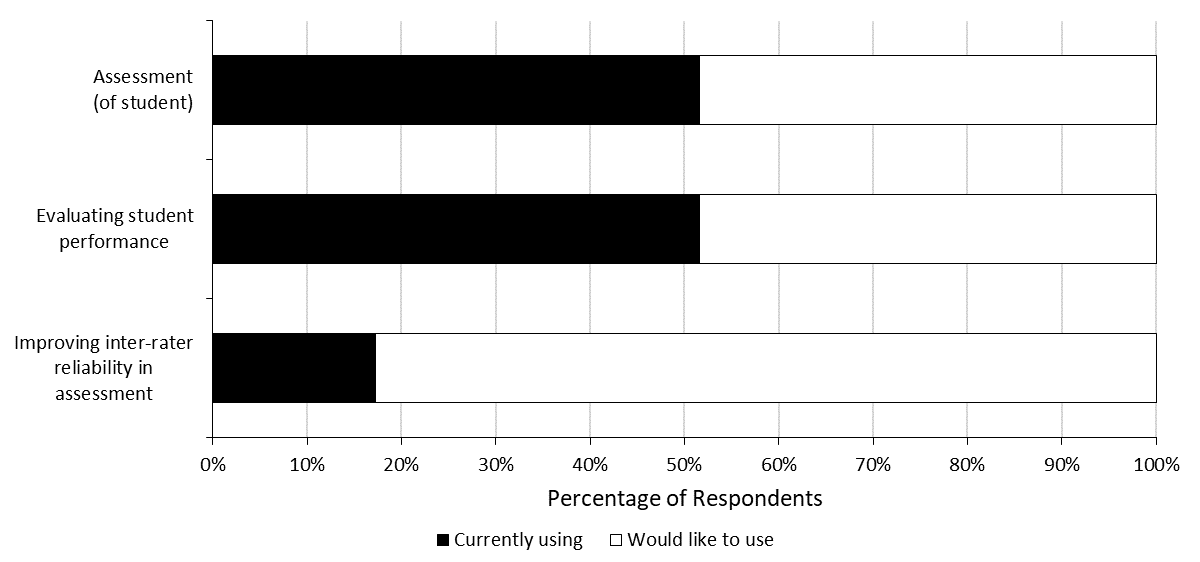


**Figure E1. Plot of respondent percentage data from Table E1 above. Ranked from top to bottom according to decreasing current use of activity (and therefore increasing wish to use activity).**

**Section F – Question 17
*[Answered only by those who self-identified as ‘New Adopters’ in Q12, n ≈ 34]***

| **Q17 - Indicate if you are currently using simulation, or would like to use simulation in the following areas related to work placements:** | | | | | | | | |  |
| --- | --- | --- | --- | --- | --- | --- | --- | --- | --- |
|  | Address issues  raised by placement  providers | Easing the burden on  placement supervisors | Increasing program  capacity | Substitute simulation  for practice experience | Uncommon client  situations, experiences  or events | Unachievable practical  situations experiences  or events | Providing experiences  replicating the workplace | Standardising experience  (all students receive  similar experience) | |
| Question Label | Q17_4 | Q17_6 | Q17_1 | Q17_7 | Q17_3 | Q17_2 | Q17_8 | Q17_5 | |
|  | Number of Respondents | | | | | | | | |
| Currently using | 3 | 4 | 6 | 7 | 10 | 11 | 12 | 12 | |
| Would like to use | 24 | 23 | 21 | 16 | 20 | 19 | 19 | 16 | |
| Total n | 27 | 27 | 27 | 23 | 30 | 30 | 31 | 28 | |
|  | Percentage of Respondents | | | | | | | | |
| Currently using | 11.1 | 14.8 | 22.2 | 30.4 | 33.3 | 36.7 | 38.7 | 42.9 | |
| Would like to use | 88.9 | 85.2 | 77.8 | 69.6 | 66.7 | 63.3 | 61.3 | 57.1 | |
| Total % | 100 | 100 | 100 | 100 | 100 | 100 | 100 | 100 | |

**Table F1. Number and percentage of respondents resolved according certain simulation activities related to work placements they are either currently using or would like to use.**


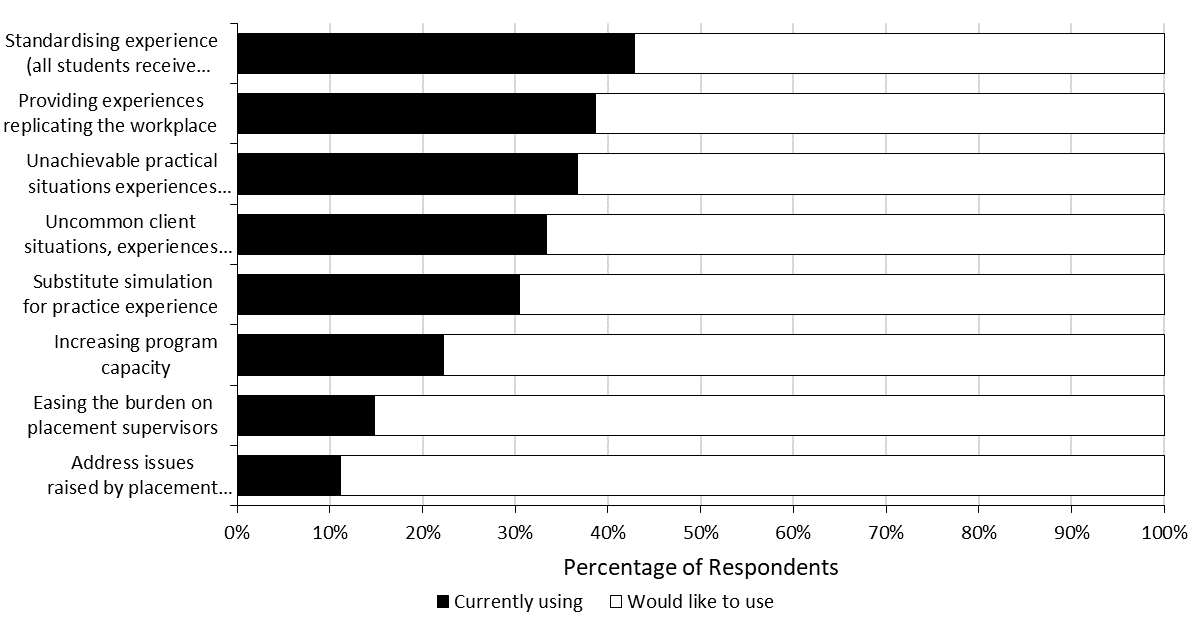


**Figure F1. Plot of respondent percentage data from Table F1 above. Ranked from top to bottom according to decreasing current use of activity (and therefore increasing wish to use activity).**

| **Q18 - Rate your level of agreement with the following barriers to integrating simulation into your program:** | | | | | | | | | | | | | |  | | |  |  |
| --- | --- | --- | --- | --- | --- | --- | --- | --- | --- | --- | --- | --- | --- | --- | --- | --- | --- | --- |
|  | Integrating simulation into  my program will diminish quality. | Students won’t react  well to simulation | I do not believe client  presentation can be realistically  portrayed using simulation. | These methods are not  suited to my program | I feel that using new  methods is risky | Teaching innovation is a  relatively low priority  in my program | The culture of my work area  does not support integration of  simulation. | There are no simulation resources  available for my program | I am not aware of available  simulation methods and products | I am satisfied with the  teaching methods used | There is limited support  available (e.g. technical or  admin.) for new methods | There are limited simulation  resources to enable use of  simulation methods | I have limited time available  for teaching innovation  and development | | |  |  |  |
| Question Label | Q18_11 | Q18_8 | Q18_12 | Q18_6 | Q18_2 | Q18_9 | Q18_13 | Q18_7 | Q18_3 | Q18_4 | Q18_10 | Q18_5 | Q18_1 | | |  |  |  |
|  | Number of Respondents | | | | | | | | | | | | | |  |  |  |  |
| Strong agree | 0 | 0 | 0 | 0 | 0 | 0 | 1 | 0 | 2 | 1 | 8 | 7 | 10 | | |  |  |  |
| Agree | 1 | 0 | 1 | 1 | 1 | 6 | 5 | 6 | 10 | 9 | 14 | 17 | 9 | | |  |  |  |
| Neutral | 1 | 1 | 3 | 4 | 5 | 4 | 4 | 7 | 5 | 14 | 6 | 3 | 12 | | |  |  |  |
| Disagree | 16 | 24 | 21 | 23 | 22 | 15 | 16 | 14 | 15 | 10 | 5 | 7 | 2 | | |  |  |  |
| Strong disagree | 16 | 9 | 9 | 6 | 6 | 9 | 8 | 7 | 2 | 0 | 1 | 0 | 1 | | |  |  |  |
| Total n | 34 | 34 | 34 | 34 | 34 | 34 | 34 | 34 | 34 | 34 | 34 | 34 | 34 | | |  |  |  |
|  | Percentage of Respondents | | | | | | | | | | | | | |  |  |  |  |
| Strong agree | 0.0 | 0.0 | 0.0 | 0.0 | 0.0 | 0.0 | 2.9 | 0.0 | 5.9 | 2.9 | 23.5 | 20.6 | 29.4 | | |  |  |  |
| Agree | 2.9 | 0.0 | 2.9 | 2.9 | 2.9 | 17.6 | 14.7 | 17.6 | 29.4 | 26.5 | 41.2 | 50.0 | 26.5 | | |  |  |  |
| Neutral | 2.9 | 2.9 | 8.8 | 11.8 | 14.7 | 11.8 | 11.8 | 20.6 | 14.7 | 41.2 | 17.6 | 8.8 | 35.3 | | |  |  |  |
| Disagree | 47.1 | 70.6 | 61.8 | 67.6 | 64.7 | 44.1 | 47.1 | 41.2 | 44.1 | 29.4 | 14.7 | 20.6 | 5.9 | | |  |  |  |
| Strong disagree | 47.1 | 26.5 | 26.5 | 17.6 | 17.6 | 26.5 | 23.5 | 20.6 | 5.9 | 0.0 | 2.9 | 0.0 | 2.9 | | |  |  |  |
| Total % | 100 | 100 | 100 | 100 | 100 | 100 | 100 | 100 | 100 | 100 | 100 | 100 | 100 | | |  |  |  |

**Table G1. Number and percentage of respondents resolved according to their perceptions of barriers to integrating simulation into their programs. (Likert scale 1 – 5: Strongly disagree – Strongly agree).**


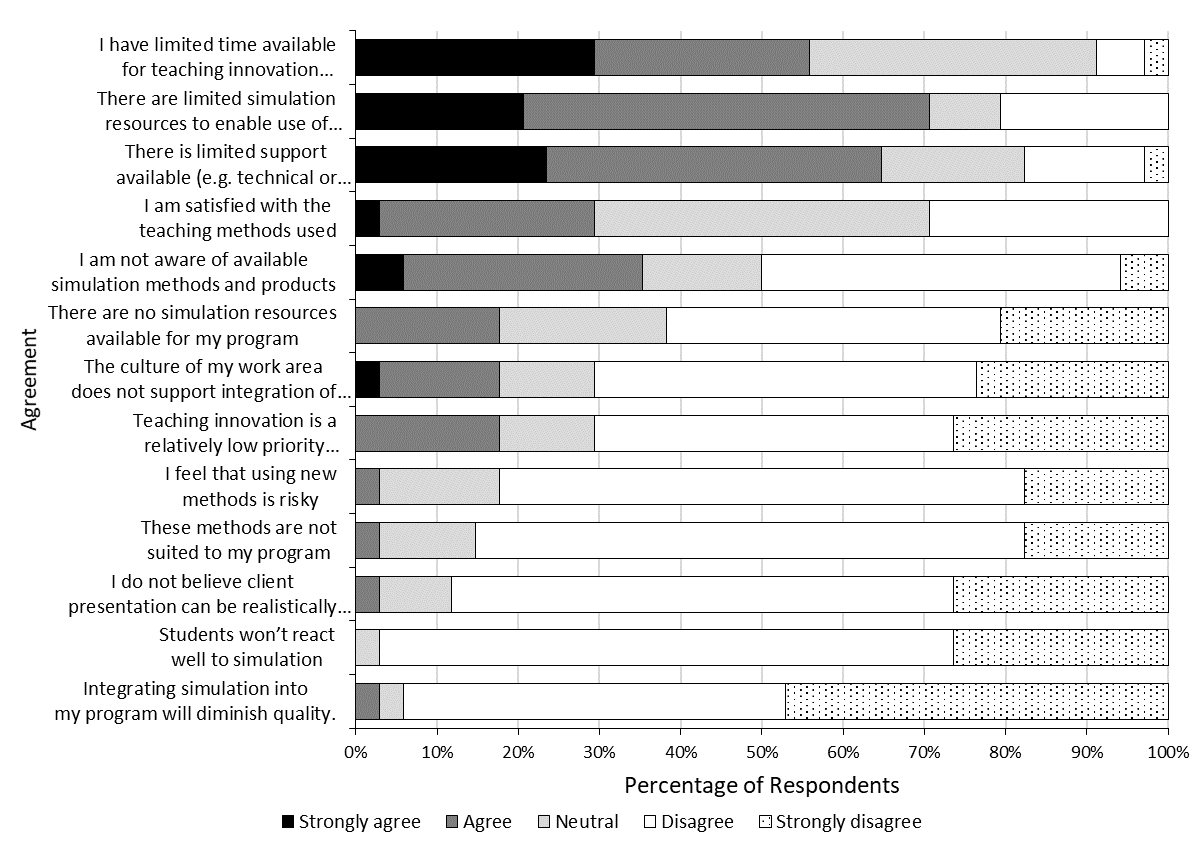


**Figure G1.** **Plot of respondent percentage data from Table G1 above. Ranked from top to bottom according to decreasing significance of barriers to integration of simulation in program.**

***‘Technicians’***

| **Q19_1 - Rank your confidence in the following items:** | | | | | | | | |  | | |  |
| --- | --- | --- | --- | --- | --- | --- | --- | --- | --- | --- | --- | --- |
| Your Confidence in | Developing  business cases | Developing  budgets | Moulage | Operating AV equip.  for simulation | Manikin  technology | Healthcare  terminology | Laptops and  simulator software | Medical equipment  and consumable items | | |  |  |
| Question Label | Q19_1_2 | Q19_1_1 | Q19_1_7 | Q19_1_8 | Q19_1_5 | Q19_1_3 | Q19_1_4 | Q19_1_6 | | |  |  |
|  | Number of Respondents | | | | | | | | |  |  |  |
| Extremely confident | 3 | 5 | 5 | 6 | 11 | 12 | 11 | 12 | | |  |  |
| Mostly confident | 4 | 2 | 12 | 9 | 9 | 8 | 9 | 10 | | |  |  |
| Confident | 4 | 5 | 2 | 7 | 4 | 3 | 5 | 2 | | |  |  |
| Somewhat confident | 7 | 7 | 3 | 3 | 0 | 2 | 0 | 1 | | |  |  |
| Not confident | 4 | 4 | 2 | 0 | 1 | 0 | 0 | 0 | | |  |  |
| Total n | 22 | 23 | 24 | 25 | 25 | 25 | 25 | 25 | | |  |  |
|  | Percentage of Respondents | | | | | | | | |  |  |  |
| Extremely confident | 13.6 | 21.7 | 20.8 | 24.0 | 44.0 | 48.0 | 44.0 | 48.0 | | |  |  |
| Mostly confident | 18.2 | 8.7 | 50.0 | 36.0 | 36.0 | 32.0 | 36.0 | 40.0 | | |  |  |
| Confident | 18.2 | 21.7 | 8.3 | 28.0 | 16.0 | 12.0 | 20.0 | 8.0 | | |  |  |
| Somewhat confident | 31.8 | 30.4 | 12.5 | 12.0 | 0.0 | 8.0 | 0.0 | 4.0 | | |  |  |
| Not confident | 18.2 | 17.4 | 8.3 | 0.0 | 4.0 | 0.0 | 0.0 | 0.0 | | |  |  |
| Total % | 100 | 100 | 100 | 100 | 100 | 100 | 100 | 100 | | |  |  |

**Table H1. Number and percentage of respondents resolved according to their assigned confidence in certain simulation related areas of expertise. (Likert scale 1 – 5: Not at all confident – Extremely confident).**

| **Q19_2 - Rank the importance of the following items** | | | | | | | | |
| --- | --- | --- | --- | --- | --- | --- | --- | --- |
| The importance of | Moulage | Developing  budgets | Healthcare  terminology | Developing  business cases | Manikin  technology | Laptops and  simulator software | Medical equipment  and consumable items | Operating AV equip. for simulation |
| Question Label | Q19_2_7 | Q19_2_1 | Q19_2_3 | Q19_2_2 | Q19_2_5 | Q19_2_4 | Q19_2_6 | Q19_2_8 |
|  | Number of Respondents | | | | | | | |
| Extremely import. | 6 | 12 | 12 | 12 | 13 | 15 | 14 | 13 |
| Moderately import. | 10 | 6 | 8 | 4 | 9 | 6 | 8 | 10 |
| Somewhat import. | 5 | 2 | 4 | 4 | 2 | 3 | 2 | 1 |
| Least Important | 3 | 0 | 0 | 0 | 1 | 1 | 1 | 1 |
| Not at all important | 0 | 2 | 1 | 1 | 0 | 0 | 0 | 0 |
| Total n | 24 | 22 | 25 | 21 | 25 | 25 | 25 | 25 |
|  | Percentage of Respondents | | | | | | | |
| Extremely import. | 25.0 | 54.5 | 48.0 | 57.1 | 52.0 | 60.0 | 56.0 | 52.0 |
| Moderately import. | 41.7 | 27.3 | 32.0 | 19.0 | 36.0 | 24.0 | 32.0 | 40.0 |
| Somewhat import. | 20.8 | 9.1 | 16.0 | 19.0 | 8.0 | 12.0 | 8.0 | 4.0 |
| Least Important | 12.5 | 0.0 | 0.0 | 0.0 | 4.0 | 4.0 | 4.0 | 4.0 |
| Not at all important | 0.0 | 9.1 | 4.0 | 4.8 | 0.0 | 0.0 | 0.0 | 0.0 |
| Total % | 100 | 100 | 100 | 100 | 100 | 100 | 100 | 100 |

**Table H2. Number and percentage of respondents resolved according to their assigned importance to certain simulation related areas of expertise. (Likert scale 1 – 5: Not at all important – Extremely important).**

| Your Confidence in . . . | Developing budgets | Developing business cases | Healthcare terminology | Laptops and simulator software | Manikin technology | Medical equip. and consumables | Moulage | Operating AV equipment for sim. |
| --- | --- | --- | --- | --- | --- | --- | --- | --- |
| Developing budgets | 1 | .950 | -.136 | .096 | .161 | .070 | -.179 | .191 |
| Developing business cases | .950 | 1 | -.096 | .039 | .011 | .053 | -.188 | .212 |
| Healthcare terminology | -.136 | -.096 | 1 | .141 | .224 | .619 | .591 | -.111 |
| Laptops and simulator software | .096 | .039 | .141 | 1 | .812 | .356 | .021 | .562 |
| Manikin technology | .161 | .011 | .224 | .812 | 1 | .418 | .131 | .467 |
| Medical equip. and consumables | .070 | .053 | .619 | .356 | .418 | 1 | .637 | -.135 |
| Moulage | -.179 | -.188 | .591 | .021 | .131 | .637 | 1 | -.130 |
| Operating AR equipment for sim. | .191 | .212 | -.111 | .562 | .467 | -.135 | -.130 | 1 |
|  |  |  |  |  |  |  |  |  |
| Mean Spearman's rho | .207 |  |  |  |  |  |  |  |

**Table H3. Correlation matrix for the eight items in the (Q19_1) assigned confidence scale, showing Spearman’s** ρ **as the correlation coefficient. The mean value, *ρ* = 0.21, indicates a modest degree of overlap between the scale items.**

| The Importance of . . . | Developing budgets | Developing business cases | Healthcare terminology | | Laptops and simulator software | | Manikin technology | | Medical equip. and consumables | | Moulage | | Operating AV equip. for sim. | |  |  |
| --- | --- | --- | --- | --- | --- | --- | --- | --- | --- | --- | --- | --- | --- | --- | --- | --- |
| Developing budgets | 1 | .747 | -.054 | | -.368 | | -.288 | | .190 | | -.063 | | -.121 | |  |  |
| Developing business cases | .747 | 1 | -.105 | | -.252 | | -.040 | | -.087 | | -.069 | | .151 | |  |  |
| Healthcare terminology | -.054 | -.105 | 1 | | .071 | | .352 | | .597 | | .679 | | .189 | |  |  |
| Laptops and simulator software | -.368 | -.252 | .071 | | 1 | | .771 | | .132 | | -.017 | | .172 | |  |  |
| Manikin technology | -.288 | -.040 | .352 | | .771 | | 1 | | .165 | | .119 | | .270 | |  |  |
| Medical equip. and consumables | .190 | -.087 | .597 | | .132 | | .165 | | 1 | | .467 | | .026 | |  |  |
| Moulage | -.063 | -.069 | .679 | | -.017 | | .119 | | .467 | | 1 | | .259 | |  |  |
| Operating AR equip. for sim. | -.121 | .151 | .189 | | .172 | | .270 | | .026 | | .259 | | 1 | |  |  |
|  |  |  |  | |  | |  | |  | |  | |  | | |  |
| Mean Spearman’s rho | .139 |  | |  | |  | |  | |  | |  | |  | | |

**Table H3. Correlation matrix for the eight items in the (Q19_2) assigned importance-scale, showing Spearman’s** ρ **as the correlation coefficient. The mean value, *ρ* = 0.14, indicates a low degree of overlap between the scale items.**


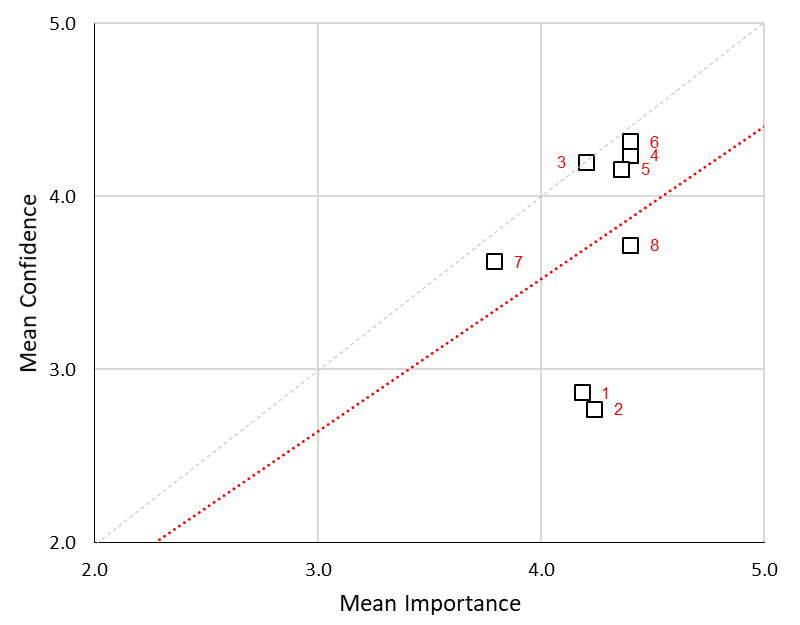


**Figure H3.** **Scatterplot of Mean Confidence *vs*. Mean Importance for the eight items in the Q19 scale.** **The red dotted line is the OLS (ordinary least-squares) line of best fit to the data (in the form *y* = m*x*).** **The grey dotted line is the 1:1 line, (i.e. where Mean Confidence = Mean Importance). The plot labels 1 – 8 correspond to Q19_*_1 to Q19_*_8; * is 1 or 2.**

| **Test of Normality** |  | |  | |  | |  |
| --- | --- | --- | --- | --- | --- | --- | --- |
|  | | Shapiro-Wilk | | | | | |
|  |  | Statistic | | df | | Sig. | |
| Your confidence in – Overall (Q19_1) | | .982 | | 25 | | .926 | |
| The importance of – Overall (Q19_2) | | .893***** | | 25 | | .013 | |

**Table H5.** **Results of the (Shapiro-Wilk) test for normality on the aggregated mean responses to Q19_1_1-8 and Q19_2_1-8, respectively. For Q19_2, the data departs significantly (p < 0.05) from normality. This indicates that a nonparametric test for differences between the matched mean responses should be used (e.g. the Wilcoxon Signed Rank Test).**

| **Wilcoxon Signed Rank Test** | |  |
| --- | --- | --- |
|  | [The importance of – Overall (Q19_2)] -  [Your confidence in – Overall (Q19_1)] | |
| Z | -2.744****** | |
| Asymp. Sig. (2-tailed) | .006 | |

**Table H6.** **Results of the Wilcoxon Signed Rank Test on the matched differences between aggregated mean responses to Q19_1 and Q19_2, respectively. The responses are different to a significant degree, at the *p* < 0.01 level.**

| **Reliability Statistics** | | |  |  |  |
| --- | --- | --- | --- | --- | --- |
| N of Items | Cronbach's Alpha | | |  |  |
|  | Confidence | Importance | |  |  |
| 8 | .643 | .637 | |  |  |
|  |  |  | |  |  |
| **Item-Total Statistics** |  |  | |  |  |
| Item | Cronbach's Alpha if Item Deleted | Cronbach's Alpha if Item Deleted | |  |  |
| Developing budgets | .596 | .708 | |  |  |
| Developing business cases | .607 | .669 | |  |  |
| Healthcare terminology | .605 | .518 | |  |  |
| Laptops and simulator software | .599 | .611 | |  |  |
| Manikin technology | .583 | .566 | |  |  |
| Medical equipment and consumable items | .580 | .533 | |  |  |
| Moulage | .697 | .558 | |  |  |
| Operating audio-visual equipment for simulation | .616 | .636 | |  |  |

**Table H6. Results of running a Reliability Analysis on the 8-item Q19 scale for both Confidence and Importance, using Cronbach’s Alpha.**

| **Q20_1 – Rank your confidence in the following items:** | | | | | | | | | | | |
| --- | --- | --- | --- | --- | --- | --- | --- | --- | --- | --- | --- |
| Your Confidence in | Managing asset registers  including repairs maintenance  loans etc. | Programming different  simulator makes and models | Developing and maintaining  schedules for maintenance | Developing and implementing  processes for equipment replacement | Trouble shooting  equipment failure | Developing and maintaining  relationships with vendors | Managing inventories of  simulation equipment | Managing inventories  of consumables | Coordinating transport  of resources | Running simulators in  simulation activities | |
| Question Label | Q20_1_5 | Q20_1_8 | Q20_1_4 | Q20_1_2 | Q20_1_10 | Q20_1_3 | Q20_1_7 | Q20_1_6 | Q20_1_1 | Q20_1_9 | |
|  | Number of Respondents | | | | | | | | | |  |
| Extreme confident | 3 | 5 | 5 | 3 | 8 | 7 | 7 | 10 | 7 | 9 | |
| Mostly confident | 8 | 6 | 6 | 8 | 9 | 9 | 9 | 5 | 6 | 10 | |
| Confident | 8 | 9 | 8 | 8 | 5 | 7 | 7 | 8 | 5 | 5 | |
| Somewhat confident | 3 | 4 | 4 | 4 | 2 | 1 | 1 | 1 | 1 | 1 | |
| Not Confident | 1 | 1 | 1 | 0 | 1 | 0 | 0 | 0 | 0 | 0 | |
| Total n | 23 | 25 | 24 | 23 | 25 | 24 | 24 | 24 | 19 | 25 | |
|  | Percentage of Respondents | | | | | | | | | |  |
| Extremely confident | 13.0 | 20.0 | 20.8 | 13.0 | 32.0 | 29.2 | 29.2 | 41.7 | 36.8 | 36.0 | |
| Mostly confident | 34.8 | 24.0 | 25.0 | 34.8 | 36.0 | 37.5 | 37.5 | 20.8 | 31.6 | 40.0 | |
| Confident | 34.8 | 36.0 | 33.3 | 34.8 | 20.0 | 29.2 | 29.2 | 33.3 | 26.3 | 20.0 | |
| Somewhat confident | 13.0 | 16.0 | 16.7 | 17.4 | 8.0 | 4.2 | 4.2 | 4.2 | 5.3 | 4.0 | |
| Not Confident | 4.3 | 4.0 | 4.2 | 0.0 | 4.0 | 0.0 | 0.0 | 0.0 | 0.0 | 0.0 | |
| Total % | 100 | 100 | 100 | 100 | 100 | 100 | 100 | 100 | 100 | 100 | |

**Table J1. Number and percentage of respondents resolved according to their assigned confidence in certain simulation related areas of expertise. (Likert scale 1 – 5: Not at all confident – Extremely confident).**

| **Q20_2 – Rank the importance of the following activities:** | | | | | | | | | | |
| --- | --- | --- | --- | --- | --- | --- | --- | --- | --- | --- |
| The Importance of | Coordinating transport  of resources | Programming different  simulator makes and models | Developing and maintaining  relationships with vendors | Managing inventories of  consumables | Developing and implementing  processes for equipment  replacement | Managing inventories of  simulation equipment | Managing asset registers including  repairs maintenance loans etc. | Developing and maintaining  schedules for maintenance | Running simulators in  simulation activities | Trouble shooting  equipment failure |
| Question Label | Q20_2_1 | Q20_2_8 | Q20_2_3 | Q20_2_6 | Q20_2_2 | Q20_2_7 | Q20_2_5 | Q20_2_4 | Q20_2_9 | Q20_2_10 |
|  | Number of Respondents | | | | | | | | | |
| Extremely important | 6 | 9 | 9 | 13 | 11 | 11 | 12 | 15 | 19 | 19 |
| Moderately important | 5 | 12 | 11 | 7 | 9 | 12 | 10 | 8 | 4 | 4 |
| Somewhat important | 6 | 2 | 2 | 4 | 2 | 2 | 1 | 1 | 2 | 2 |
| Least important | 2 | 2 | 2 | 1 | 1 | 0 | 1 | 1 | 0 | 0 |
| Not at all important | 0 | 0 | 0 | 0 | 0 | 0 | 0 | 0 | 0 | 0 |
| Total n | 19 | 25 | 24 | 25 | 23 | 25 | 24 | 25 | 25 | 25 |
|  | Percentage of Respondents | | | | | | | | | |
| Extremely important | 31.6 | 36.0 | 37.5 | 52.0 | 47.8 | 44.0 | 50.0 | 60.0 | 76.0 | 76.0 |
| Moderately important | 26.3 | 48.0 | 45.8 | 28.0 | 39.1 | 48.0 | 41.7 | 32.0 | 16.0 | 16.0 |
| Somewhat important | 31.6 | 8.0 | 8.3 | 16.0 | 8.7 | 8.0 | 4.2 | 4.0 | 8.0 | 8.0 |
| Least important | 10.5 | 8.0 | 8.3 | 4.0 | 4.3 | 0.0 | 4.2 | 4.0 | 0.0 | 0.0 |
| Not at all important | 0.0 | 0.0 | 0.0 | 0.0 | 0.0 | 0.0 | 0.0 | 0.0 | 0.0 | 0.0 |
| Total % | 100 | 100 | 100 | 100 | 100 | 100 | 100 | 100 | 100 | 100 |

**Table J2. Number and percentage of respondents resolved according to their assigned Importance to certain simulation related areas of expertise. (Likert scale 1 – 5: Not at all important – Extremely important).**

| Your Confidence in | Coordinating transport of resources | | Developing and implementing processes for equipment replacement | Developing and maintaining relationships with vendors | Developing and maintaining schedules for maintenance | Managing asset registers including repairs maintenance loans etc. | Managing inventories of consumables | Managing inventories of simulation equipment | Programming different simulator makes and models | Running simulators in simulation activities | Trouble shooting equipment failure |
| --- | --- | --- | --- | --- | --- | --- | --- | --- | --- | --- | --- |
| Coordinating transport of resources | 1 | | .489 | .244 | .185 | .135 | .453 | .344 | .269 | .320 | .195 |
| Developing and implementing processes for equipment replacement | .489 | | 1 | .762 | .752 | .719 | .513 | .510 | .425 | .334 | .389 |
| Developing and maintaining relationships with vendors | .244 | | .762 | 1 | .775 | .721 | .463 | .562 | .654 | .509 | .566 |
| Developing and maintaining schedules for maintenance | .185 | | .752 | .775 | 1 | .831 | .592 | .514 | .655 | .549 | .549 |
| Managing asset registers including repairs maintenance loans etc. | .135 | | .719 | .721 | .831 | 1 | .730 | .754 | .492 | .348 | .283 |
| Managing inventories of consumables | .453 | | .513 | .463 | .592 | .730 | 1 | .900 | .482 | .446 | .335 |
| Managing inventories of simulation equipment | .344 | | .510 | .562 | .514 | .754 | .900 | 1 | .436 | .310 | .260 |
| Programming different simulator makes and models | .269 | | .425 | .654 | .655 | .492 | .482 | .436 | 1 | .616 | .706 |
| Running simulators in simulation activities | .320 | | .334 | .509 | .549 | .348 | .446 | .310 | .616 | 1 | .569 |
| Trouble shooting equipment failure | .195 | | .389 | .566 | .549 | .283 | .335 | .260 | .706 | .569 | 1 |
|  | |  |  |  |  |  |  |  |  |  |  |
| Mean Spearman's rho | .503 | |  |  |  |  |  |  |  |  |  |

**Table J3. Correlation matrix for the ten items in the (Q20_1) assigned confidence scale, showing Spearman’s** ***ρ* as the correlation coefficient. The mean value, *ρ* = 0.50, indicates a moderate degree of overlap between the scale items.**

| The Importance of | | | Coordinating transport of resources | | Developing and implementing processes for equipment replacement | | Developing and maintaining relationships with vendors | | Developing and maintaining schedules for maintenance | | Managing asset registers including repairs maintenance loans etc. | | Managing inventories of consumables | | Managing inventories of simulation equipment | | Programming different simulator makes and models | | Running simulators in simulation activities | | Trouble shooting equipment failure |
| --- | --- | --- | --- | --- | --- | --- | --- | --- | --- | --- | --- | --- | --- | --- | --- | --- | --- | --- | --- | --- | --- |
| Coordinating transport of resources | | | 1 | | .414 | | .534 | | .374 | | .256 | | .491 | | .553 | | .471 | | .358 | | .405 |
| Developing and implementing processes for equipment replacement | | | .414 | | 1 | | .245 | | .376 | | .497 | | .261 | | .077 | | .365 | | .363 | | .666 |
| Developing and maintaining relationships with vendors | | | .534 | | .245 | | 1 | | .587 | | .474 | | .419 | | .662 | | .424 | | .560 | | .588 |
| Developing and maintaining schedules for maintenance | | | .374 | | .376 | | .587 | | 1 | | .387 | | .423 | | .403 | | .502 | | .299 | | .578 |
| Managing asset registers including repairs maintenance loans etc. | | | .256 | | .497 | | .474 | | .387 | | 1 | | .351 | | .235 | | .353 | | .340 | | .536 |
| Managing inventories of consumables | | | .491 | | .261 | | .419 | | .423 | | .351 | | 1 | | .424 | | .258 | | .279 | | .176 |
| Managing inventories of simulation equipment | | | .553 | | .077 | | .662 | | .403 | | .235 | | .424 | | 1 | | .510 | | .504 | | .352 |
| Programming different simulator makes and models | | | .471 | | .365 | | .424 | | .502 | | .353 | | .258 | | .510 | | 1 | | .563 | | .684 |
| Running simulators in simulation activities | | | .358 | | .363 | | .560 | | .299 | | .340 | | .279 | | .504 | | .563 | | 1 | | .805 |
| Trouble shooting equipment failure | | | .405 | | .666 | | .588 | | .578 | | .536 | | .176 | | .352 | | .684 | | .805 | | 1 |
|  |  |  | |  | |  | |  | |  | |  | |  | |  | |  | |  |  |
| Mean Spearman’s rho | | | .431 | |  | |  | |  | |  | |  | |  | |  | |  | |  |

**Table J4. Correlation matrix for the ten items in the (Q20_2) assigned importance scale, showing Spearman’s *ρ* as the correlation coefficient. The mean value, *ρ* = 0.43, indicates a moderate level of overlap between the scale items.**


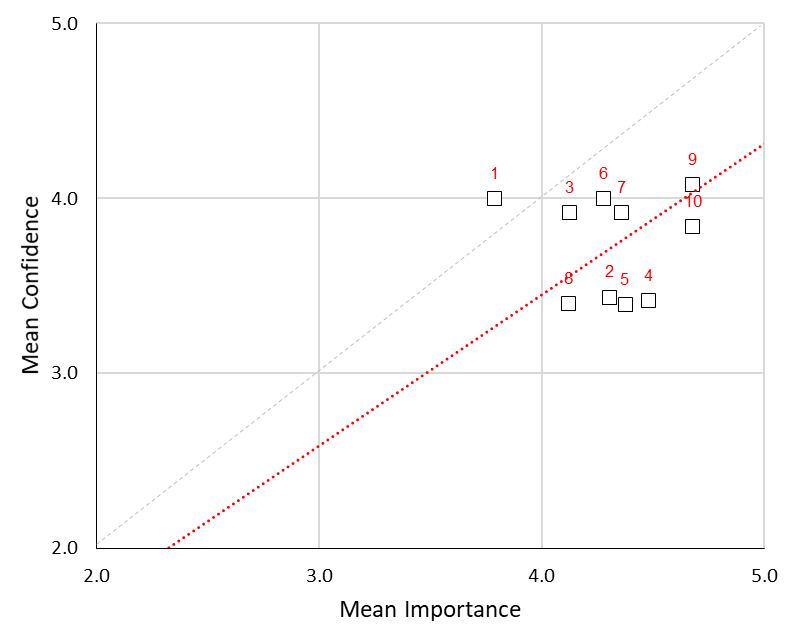


**Figure J3****. Scatterplot of Mean Confidence *vs*. Mean Importance for the ten items in the Q20 scale. The red dotted line is the OLS line (*y* = m*x*) of best fit to the data.** **The grey dotted line is the 1:1 line. The plot labels 1 – 10 correspond to Q20_*_1 to Q20_*_10.**

| **Test of Normality** |  | | |  | |  |  |
| --- | --- | --- | --- | --- | --- | --- | --- |
|  | | Shapiro-Wilk | | | | | |
|  |  | Statistic | df | | Sig. | | |
| Your confidence in – Overall (Q20_1) | | .951 | 25 | | .265 | | |
| The importance of – Overall (Q20_2) | | .899***** | 25 | | .017 | | |

**Table J5. Results of the test for normality on the aggregated mean responses to Q20_1_1-10 and Q20_2_1-10, respectively. For Q20_2,** **the data departs significantly (*p* < 0.05) from normality. This indicates that a nonparametric test for differences between the matched mean responses should be used (e.g. the Wilcoxon Signed Rank Test).**

| **Wilcoxon Signed Rank Test** | |  | |
| --- | --- | --- | --- |
|  | [The importance of – Overall (Q20_2)] -  [Your confidence in – Overall (Q20_1)] | |  |
| Z | -2.602****** | |  |
| Asymp. Sig. (2-tailed) | .009 | |  |

**Table J6. Results of the Wilcoxon Signed Rank Test on the matched differences between aggregated mean responses to Q20_1 and Q20_2, respectively.** **The responses are different to a significant degree, at the *p* < 0.01 level.**

| **Reliability Statistics** | | |
| --- | --- | --- |
|  |  |  |
| N of Items | Cronbach’s Alpha | |
|  | Confidence | Importance |
| 10 | .905 | .908 |
|  |  |  |
| **Item-Total Statistics** |  |  |
| Item | Cronbach's Alpha if Item Deleted | Cronbach's Alpha if Item Deleted |
| Coordinating transport of resources | .914 | .911 |
| Develop.& and implem. processes for equip. replace | .895 | .900 |
| Develop. & maintain relationships with vendors | .891 | .888 |
| Develop. and maintain schedules for maintenance | .889 | .893 |
| Manag. asset registers incl. repairs maintenance loans | .889 | .899 |
| Manag. inventories of consumables | .888 | .899 |
| Manag. inventories of simulation equipment | .889 | .908 |
| Program. different simulator makes and models | .890 | .891 |
| Running simulators in simulation activities | .906 | .904 |
| Trouble shooting equipment failure | .904 | .891 |

**Table J7.** **Results of running a Reliability Analysis on the 10-item Q20 scale for both Confidence and Importance, using Cronbach’s Alpha.**

| **Q21_1 - Rank your confidence in preparing an environment for simulation sessions using:** | | | | | | |  |
| --- | --- | --- | --- | --- | --- | --- | --- |
| Your Confidence in | High technology manikin  with software | Developing health  records | Creating clinically realistic learning environments using . . . | Appropriate use of  medical equipment  and consumables | Medium technology  manikins and control devices | Task trainers | |
| Question Label | Q21_1_4 | Q21_1_3 | Q21_1_2 | Q21_1_1 | Q21_1_5 | Q21_1_6 | |
|  | Number | | | | | | |
| Extremely confident | 7 | 10 | 11 | 10 | 11 | 14 | |
| Mostly confident | 10 | 9 | 8 | 11 | 9 | 8 | |
| Confident | 6 | 0 | 4 | 2 | 5 | 3 | |
| Somewhat confident | 2 | 2 | 2 | 2 | 0 |  | |
| Not Confident | 0 | 3 | 0 | 0 | 0 | 0 | |
| Total n | 25 | 24 | 25 | 25 | 25 | 25 | |
|  | Percentage | | | | | | |
| Extremely confident | 28.0 | 41.7 | 44.0 | 40.0 | 44.0 | 56.0 | |
| Mostly confident | 40.0 | 37.5 | 32.0 | 44.0 | 36.0 | 32.0 | |
| Confident | 24.0 | 0.0 | 16.0 | 8.0 | 20.0 | 12.0 | |
| Somewhat confident | 8.0 | 8.3 | 8.0 | 8.0 | 0.0 | 0.0 | |
| Not Confident | 0.0 | 12.5 | 0.0 | 0.0 | 0.0 | 0.0 | |
| Total % | 100 | 100 | 100 | 100 | 100 | 100 | |

**Table K1. Number and percentage of respondents resolved according to their assigned confidence in certain activities in preparing an environment for simulation sessions. (Likert scale 1 – 5: Not at all confident – Extremely confident).**

| **Q21_2 - Rank the importance of preparing an environment for simulation sessions using:** | | | | | | |  |
| --- | --- | --- | --- | --- | --- | --- | --- |
| The importance of | High technology manikin  with software | Developing health  records | Medium technology  manikins and control devices | Task trainers | Creating clinically realistic learning environments using . . . | Appropriate use of  medical equipment  and consumables | |
| Question Label | Q21_2_4 | Q21_2_3 | Q21_2_5 | Q21_2_6 | Q21_2_2 | Q21_2_1 | |
|  | Number of Respondents | | | | | | |
| Extremely important | 9 | 10 | 9 | 13 | 13 | 15 | |
| Moderately important | 11 | 10 | 13 | 9 | 8 | 7 | |
| Somewhat important | 3 | 1 | 3 | 2 | 4 | 2 | |
| Least important | 2 | 2 | 0 | 0 |  | 1 | |
| Not at all important | 0 | 1 | 0 | 1 | 0 | 0 | |
| Total n | 25 | 24 | 25 | 25 | 25 | 25 | |
|  | Percentage of Respondents | | | | | | |
| Extremely important | 36.0 | 41.7 | 36.0 | 52.0 | 52.0 | 60.0 | |
| Moderately important | 44.0 | 41.7 | 52.0 | 36.0 | 32.0 | 28.0 | |
| Somewhat important | 12.0 | 4.2 | 12.0 | 8.0 | 16.0 | 8.0 | |
| Least important | 8.0 | 8.3 | 0.0 | 0.0 | 0.0 | 4.0 | |
| Not at all important | 0.0 | 4.2 | 0.0 | 4.0 | 0.0 | 0.0 | |
| Total % | 100 | 100 | 100 | 100 | 100 | 100 | |

**Table K2. Number and percentage of respondents resolved according to their assigned importance to certain activities in preparing an environment for simulation sessions. (Likert scale 1 – 5: Not at all important – Extremely important).**

| Your Confidence in | Appropriate use of medical equipment and consumables | Creating clinically realistic learning environments using . . . | Developing health record | High technology manikin with software | Medium technology manikins and control devices | Task trainers |
| --- | --- | --- | --- | --- | --- | --- |
| Appropriate use of medical equipment and consumables | 1 | .632 | .607 | .177 | .122 | .433 |
| Creating clinically realistic learning environments using . . . | .632 | 1 | .626 | .036 | .083 | .393 |
| Developing health records | .607 | .626 | 1 | -0.202 | -.032 | .656 |
| High technology manikin with software | .177 | .036 | -.202 | 1 | .771 | .208 |
| Medium technology manikins and control devices | .122 | .083 | -.032 | .771 | 1 | .313 |
| Task trainers | .433 | .393 | .656 | .208 | .313 | 1 |
|  | | | | | | |
| Mean Spearman's rho | .322 |  |  |  |  |  |

**Table K3. Correlation matrix for the six items in the (Q21_1) assigned confidence scale, showing Spearman’s *ρ* as the correlation coefficient. The mean value, *ρ* = 0.32, indicates a modest degree of overlap between the scale items.**

| The Importance of | Appropriate use of medical equipment and consumables | | | Creating clinically realistic learning environments using . . . | | | | Developing health records | | | High technology manikin with software | | | Medium technology manikins and control devices | | | Task trainers |
| --- | --- | --- | --- | --- | --- | --- | --- | --- | --- | --- | --- | --- | --- | --- | --- | --- | --- |
| Appropriate use of medical equipment and consumables | 1 | | | .665 | | | | .642 | | | .447 | | | .609 | | | .574 |
| Creating clinically realistic learning environments using . . . | .665 | | | 1 | | | | .617 | | | .406 | | | .346 | | | .464 |
| Developing health records | .642 | | | .617 | | | | 1 | | | .292 | | | .406 | | | .545 |
| High technology manikin with software | .447 | | | .406 | | | | .292 | | | 1 | | | .789 | | | .590 |
| Medium technology manikins and control devices | .609 | | | .346 | | | | .406 | | | .789 | | | 1 | | | .759 |
| Task trainers | .574 | | | .464 | | | | .545 | | | .590 | | | .759 | | | 1 |
|  | |  |  | |  | |  | |  | | |  | | |  |  |  |
| Mean Spearman's rho | 0.543 | | |  | |  | |  | |  | | |  | | |  |  |

**Table K4. Correlation matrix for the six items in the (Q21_2) assigned importance scale, showing Spearman’s *ρ* as the correlation coefficient. The mean value, *ρ* = 0.54, indicates a moderate degree of overlap between the scale items.**


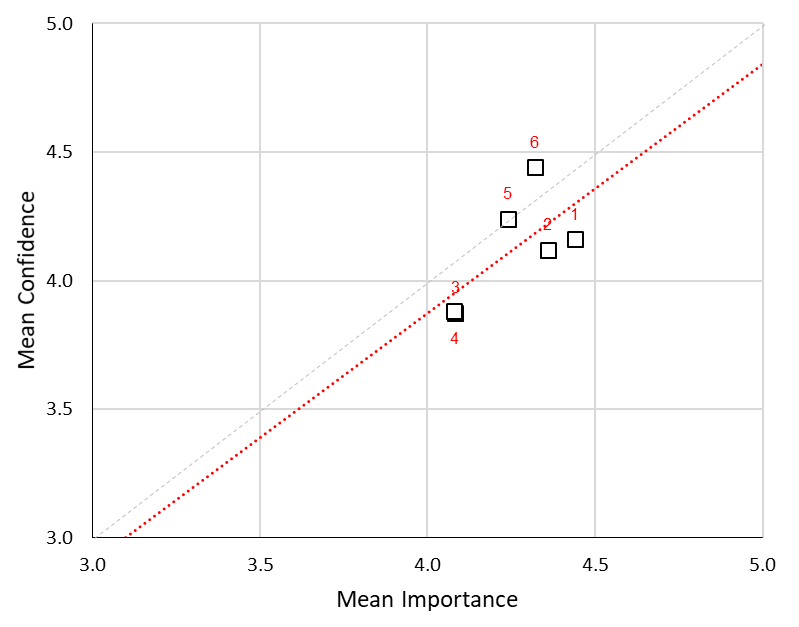


**Figure K3. Scatterplot of Mean Confidence *vs*. Mean Importance for the six items in the Q21 scale. The red dotted line is the OLS line (*y* = m*x*) of best fit to the data. The grey dotted line is the 1:1 line. The plot labels 1 – 6 correspond to Q21_*_1 to Q21_*_6).**

| **Test of Normality** | | | |  |
| --- | --- | --- | --- | --- |
|  | Shapiro-Wilk | | | |
|  | Statistic | df | Sig. | |
| Your confidence in – Overall (Q21_1) | .925 | 25 | .066 | |
| The importance of – Overall (Q21_2) | .891***** | 25 | .012 | |

**Table K5. Results of the test for normality on the aggregated mean responses to Q21_1_1-6 and Q21_2_1-6, respectively. For Q21_2, the data departs significantly (*p* < 0.05) from normality. This indicates that a nonparametric test for differences between the matched mean responses should be used (e.g. the Wilcoxon Signed Rank Test).**

| **Wilcoxon Signed Rank Test** | |  |
| --- | --- | --- |
|  | [The importance of – Overall (Q21_2)] -  [Your confidence in – Overall (Q21_1)] | |
| Z | -1.046 | |
| Asymp. Sig. (2-tailed) | .295 | |

**Table K6. Results of the Wilcoxon Signed Rank Test on the matched differences between aggregated mean responses to Q21_1 and Q21_2, respectively. The responses are not different to a significant degree, at the *p* < 0.05 level.**

| **Reliability Statistics** |  |  | |  |  |
| --- | --- | --- | --- | --- | --- |
| N of Items | | | Cronbach's Alpha | | |
|  |  |  | Confidence | | Importance |
| 6 | | | .748 | | .880 |
|  | | |  | |  |
| **Item-Total Statistics** | | |  | |  |
| Item | | | Cronbach's Alpha if Item Deleted | | Cronbach's Alpha if Item Deleted |
| Appropriate use of medical equip. and consumables | | | .644 | | .838 |
| Create. clinically real. learn. environ. using IV infusion | | | .682 | | .858 |
| Developing health records | | | .685 | | .866 |
| High technology manikin with software | | | .788 | | .883 |
| Medium technology manikins and control devices | | | .756 | | .865 |
| Task trainers | | | .682 | | .845 |

**Table K7. Results of running a Reliability Analysis on the 6-item Q21 scale for both Confidence and Importance, using Cronbach’s Alpha.**

| **Q22 - How often do you:** | | | | | | |  |
| --- | --- | --- | --- | --- | --- | --- | --- |
|  | Participate in  research regarding  simulation | Build programs  using simulator  software | Collaborate with  faculty to plan/ develop scenarios | Orientate participants  to simulators and the  simulation learning  environment | Work directly with  students/learners  in simulation | Adopt support role in simulation  activities | |
| Question Label | Q22_5 | Q22_2 | Q22_3 | Q22_4 | Q22_6 | Q22_1 | |
|  | Number of Respondents | | | | | | |
| Daily | 0 | 1 | 1 | 3 | 8 | 10 | |
| Weekly | 2 | 1 | 7 | 7 | 10 | 9 | |
| Monthly | 1 | 4 | 6 | 4 | 1 | 3 | |
| Quarterly | 3 | 4 | 2 | 7 | 0 | 0 | |
| Less than quarterly | 10 | 8 | 4 | 3 | 2 | 1 | |
| Total n | 16 | 18 | 20 | 24 | 21 | 23 | |
|  | Percentage of Respondents | | | | | | |
| Daily | 0.0 | 5.6 | 5.0 | 12.5 | 38.1 | 43.5 | |
| Weekly | 12.5 | 5.6 | 35.0 | 29.2 | 47.6 | 39.1 | |
| Monthly | 6.3 | 22.2 | 30.0 | 16.7 | 4.8 | 13.0 | |
| Quarterly | 18.8 | 22.2 | 10.0 | 29.2 | 0.0 | 0.0 | |
| Less than quarterly | 62.5 | 44.4 | 20.0 | 12.5 | 9.5 | 4.3 | |
| Total % | 100 | 100 | 100 | 100 | 100 | 100 | |

**Table L1. Number and percentage of respondents resolved according to the frequency with which engage in certain simulation related professional activities.**


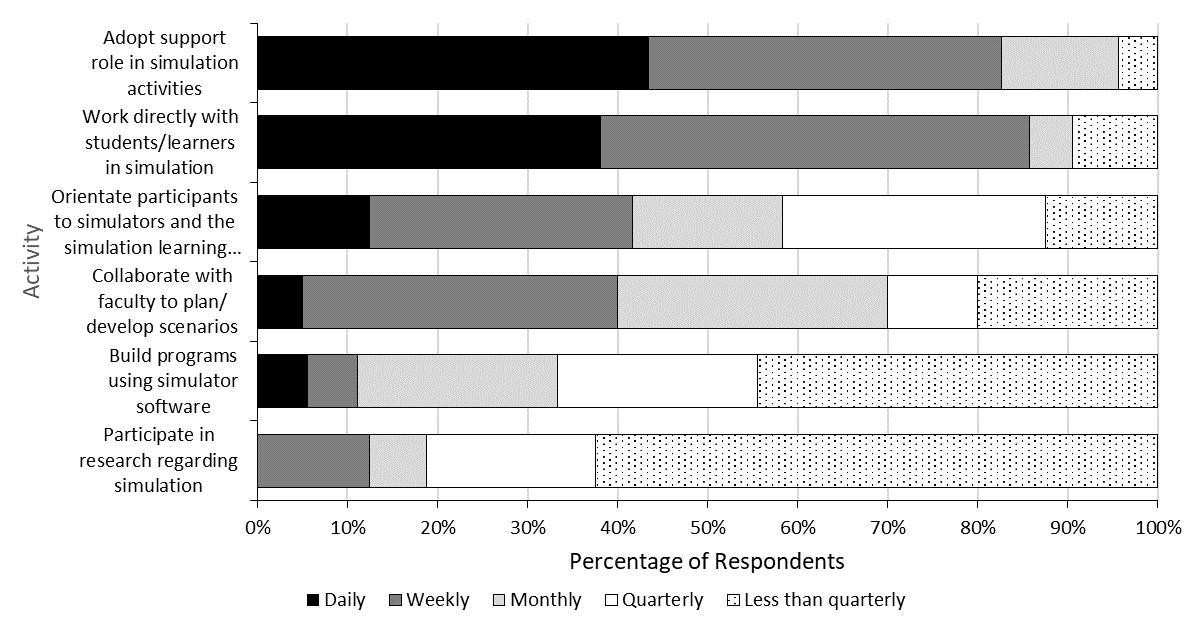


**Figure L1. Plot of respondent percentage data from Table L1 above. Ranked from top to bottom according to decreasing frequency of activity.**

***‘Simulation Users’***

| **Q27_1 - Rate your confidence in doing the following items:** | | | | | | | | | | | | |  |  |
| --- | --- | --- | --- | --- | --- | --- | --- | --- | --- | --- | --- | --- | --- | --- |
| Your Confidence in | Virtual reality- based simulation | Computer or web- based simulation | Time sequenced  simulations | Observational  sim. techniques | High technology  manikins | Immersive full- scale simulation | Hybrid  simulation | In-situ  simulation | Simul./standard.  patient simul. | Conducting pause  and discuss | Medium technology  manikins | Part task  trainer | | Low technology  manikins |
| Question Label | Q27_1_11 | Q27_1_10 | Q27_1_13 | Q27_1_12 | Q27_1_4 | Q27_1_6 | Q27_1_5 | Q27_1_8 | Q27_1_9 | Q27_1_7 | Q27_1_3 | Q27_1_1 | | Q27_1_2 |
|  | Number of Respondents | | | | | | | | | | | | | |
| Extrem. conf. | 3 | 6 | 7 | 18 | 20 | 23 | 22 | 21 | 26 | 31 | 38 | 43 | | 48 |
| Most conf. | 6 | 10 | 17 | 16 | 21 | 19 | 16 | 25 | 26 | 21 | 18 | 16 | | 14 |
| Confident | 10 | 18 | 9 | 10 | 15 | 16 | 14 | 11 | 10 | 9 | 7 | 5 | | 3 |
| Some. conf. | 17 | 14 | 11 | 9 | 8 | 3 | 5 | 5 | 2 | 1 | 2 | 3 | | 1 |
| Not conf. | 19 | 10 | 5 | 5 | 2 | 3 | 1 | 1 | 2 | 4 | 0 | 0 | | 0 |
| Total n | 55 | 58 | 49 | 58 | 66 | 64 | 58 | 63 | 66 | 66 | 65 | 67 | | 66 |
|  | Percentage of Respondents | | | | | | | | | | | | | |
| Extrem. conf. | 5.5 | 10.3 | 14.3 | 31.0 | 30.3 | 35.9 | 37.9 | 33.3 | 39.4 | 47.0 | 58.5 | 64.2 | | 72.7 |
| Most conf. | 10.9 | 17.2 | 34.7 | 27.6 | 31.8 | 29.7 | 27.6 | 39.7 | 39.4 | 31.8 | 27.7 | 23.9 | | 21.2 |
| Confident | 18.2 | 31.0 | 18.4 | 17.2 | 22.7 | 25.0 | 24.1 | 17.5 | 15.2 | 13.6 | 10.8 | 7.5 | | 4.5 |
| Some. conf. | 30.9 | 24.1 | 22.4 | 15.5 | 12.1 | 4.7 | 8.6 | 7.9 | 3.0 | 1.5 | 3.1 | 4.5 | | 1.5 |
| Not conf. | 34.5 | 17.2 | 10.2 | 8.6 | 3.0 | 4.7 | 1.7 | 1.6 | 3.0 | 6.1 | 0.0 | 0.0 | | 0.0 |
| Total % | 100 | 100 | 100 | 100 | 100 | 100 | 100 | 100 | 100 | 100 | 100 | 100 | | 100 |

**Table N1. Number and percentage of respondents resolved according to their assigned confidence in performing certain types of simulation activities. (Likert scale 1 – 5: Not confident – Extremely confident).**

| **Q27_2 – Rate the importance of the following items:** | | | | | | | | | | | | | |
| --- | --- | --- | --- | --- | --- | --- | --- | --- | --- | --- | --- | --- | --- |
| The Importance of | Virtual reality- based sim. | Computer or web- based sim. | Time sequenced  sim. | Observational  sim. techniques | High technology  manikins | Hybrid sim. | In-situ sim. | Conducting pause  and discuss | Low technology  manikins | Medium technology  manikins | Immersive full- scale sim. | Part task trainer | Simul./stand.  patient simul. |
| Question Label | Q27_2_11 | Q27_2_10 | Q27_2_13 | Q27_2_12 | Q27_2_4 | Q27_2_5 | Q27_2_8 | Q27_2_7 | Q27_2_2 | Q27_2_3 | Q27_2_6 | Q27_2_1 | Q27_2_9 |
|  | Number of Respondents | | | | | | | | | | | | |
| Extrem. import. | 6 | 8 | 12 | 21 | 27 | 21 | 27 | 35 | 36 | 32 | 32 | 37 | 37 |
| Mod. important | 8 | 11 | 14 | 19 | 21 | 23 | 17 | 20 | 21 | 26 | 21 | 22 | 22 |
| Some. important | 15 | 18 | 14 | 15 | 11 | 8 | 14 | 6 | 6 | 6 | 10 | 6 | 4 |
| Least important | 17 | 16 | 7 | 3 | 6 | 4 | 3 | 2 | 2 | 0 | 1 | 1 | 3 |
| Not important | 8 | 6 | 3 | 2 | 2 | 1 | 1 | 2 | 2 | 2 | 0 | 1 | 0 |
| Total n | 54 | 59 | 50 | 60 | 67 | 57 | 62 | 65 | 67 | 66 | 64 | 67 | 66 |
|  | Percentage of Respondents | | | | | | | | | | | | |
| Extrem. import. | 11.1 | 13.6 | 24.0 | 35.0 | 40.3 | 36.8 | 43.5 | 53.8 | 53.7 | 48.5 | 50.0 | 55.2 | 56.1 |
| Mod. important | 14.8 | 18.6 | 28.0 | 31.7 | 31.3 | 40.4 | 27.4 | 30.8 | 31.3 | 39.4 | 32.8 | 32.8 | 33.3 |
| Some. important | 27.8 | 30.5 | 28.0 | 25.0 | 16.4 | 14.0 | 22.6 | 9.2 | 9.0 | 9.1 | 15.6 | 9.0 | 6.1 |
| Least important | 31.5 | 27.1 | 14.0 | 5.0 | 9.0 | 7.0 | 4.8 | 3.1 | 3.0 | 0.0 | 1.6 | 1.5 | 4.5 |
| Not important | 14.8 | 10.2 | 6.0 | 3.3 | 3.0 | 1.8 | 1.6 | 3.1 | 3.0 | 3.0 | 0.0 | 1.5 | 0.0 |
| Total % | 100 | 100 | 100 | 100 | 100 | 100 | 100 | 100 | 100 | 100 | 100 | 100 | 100 |

**Table N2. Number and percentage of respondents resolved according to their assigned importance to performing certain types of simulation activities. (Likert scale 1 – 5: Not important – Extremely important).**

| Your Confidence in | Part task trainer | Low-tech manikins | Medium-tech manikins | High technology manikins | Hybrid simul. | Immersive full-scale simul. | Conduct pause and discuss | In-situ simul. | Simul./standard. patient simul. | Computer or web-based simul. | Virtual reality-based simul. | Observ. Simul. techniques | Time sequenced simul. |
| --- | --- | --- | --- | --- | --- | --- | --- | --- | --- | --- | --- | --- | --- |
| Part task trainer | 1 | .782 | .569 | .560 | .497 | .445 | .407 | .281 | .262 | -.026 | .058 | .396 | .426 |
| Low-tech manikins | .782 | 1 | .779 | .521 | .352 | .338 | .502 | .262 | .417 | .028 | .068 | .353 | .312 |
| Medium-tech manikins | .569 | .779 | 1 | .569 | .343 | .471 | .509 | .425 | .310 | .005 | .030 | .285 | .306 |
| High-tech manikins | .560 | .521 | .569 | 1 | .389 | .669 | .206 | .266 | .132 | -.010 | .124 | .327 | .334 |
| Hybrid simulation | .497 | .352 | .343 | .389 | 1 | .542 | .450 | .507 | .264 | .269 | .242 | .421 | .424 |
| Immersive full-scale simul. | .445 | .338 | .471 | .669 | .542 | 1 | .384 | .346 | .131 | -.004 | .080 | .266 | .332 |
| Conduct pause and discuss | .407 | .502 | .509 | .206 | .450 | .384 | 1 | .553 | .357 | .071 | .089 | .576 | .474 |
| In-situ simulation | .281 | .262 | .425 | .266 | .507 | .346 | .553 | 1 | .219 | .406 | .262 | .558 | .455 |
| Simul./standard. patient simul. | .262 | .417 | .310 | .132 | .264 | .131 | .357 | .219 | 1 | .396 | .287 | .396 | .323 |
| Computer or web-based simul. | -.026 | .028 | .005 | -.010 | .269 | -.004 | .071 | .406 | .396 | 1 | .768 | .460 | .488 |
| Virtual reality-based simul. | .058 | .068 | .030 | .124 | .242 | .080 | .089 | .262 | .287 | .768 | 1 | .454 | .582 |
| Observ. simul. techniques | .396 | .353 | .285 | .327 | .421 | .266 | .576 | .558 | .396 | .460 | .454 | 1 | .848 |
| Time sequenced simul. | .426 | .312 | .306 | .334 | .424 | .332 | .474 | .455 | .323 | .488 | .582 | .848 | 1 |
|  | |  |  |  |  |  |  |  |  |  |  |  |  |
| Mean Spearman's rho | .358 |  |  |  |  |  |  |  |  |  |  |  |  |

**Table N3. Correlation matrix for the 13 items in the (Q27_1) assigned confidence scale, showing Spearman’s *ρ* as the correlation coefficient. The mean value, *ρ* = 0.36, indicates a moderate degree of overlap between the scale items.**

| The Importance of | Part task trainer | Low-tech manikins | Medium-tech manikins | High technology manikins | Hybrid simul. | Immersive full-scale simul. | Conduct pause and discuss | In-situ simul. | Simul./standard. patient simul. | Computer or web-based simul. | Virtual reality-based simul. | Observ. Simul. techniques | Time sequenced simul. |
| --- | --- | --- | --- | --- | --- | --- | --- | --- | --- | --- | --- | --- | --- |
| Part task trainer | 1 | .612 | .441 | .330 | .197 | -.047 | .281 | .218 | .106 | .131 | .157 | .312 | .382 |
| Low-tech manikins | .612 | 1 | .382 | .194 | .218 | .041 | .455 | .411 | .174 | .291 | .360 | .437 | .444 |
| Medium-tech manikins | .441 | .382 | 1 | .588 | .316 | .221 | .246 | .205 | .027 | .171 | .032 | .220 | .041 |
| High-tech manikins | .330 | .194 | .588 | 1 | .251 | .561 | .157 | .033 | -.072 | .010 | -.140 | .207 | -.024 |
| Hybrid simulation | .197 | .218 | .316 | .251 | 1 | .084 | .332 | .223 | .362 | .202 | .006 | .218 | .259 |
| Immersive full-scale simul. | -.047 | .041 | .221 | .561 | .084 | 1 | .124 | .136 | -.012 | -.232 | -.230 | .161 | -.142 |
| Conduct pause and discuss | .281 | .455 | .246 | .157 | .332 | .124 | 1 | .347 | .286 | .478 | .464 | .566 | .441 |
| In-situ simulation | .218 | .411 | .205 | .033 | .223 | .136 | .347 | 1 | .249 | .250 | .224 | .441 | .477 |
| Simul./standard. patient simul. | .106 | .174 | .027 | -.072 | .362 | -.012 | .286 | .249 | 1 | .208 | .231 | .365 | .338 |
| Computer or web-based simul. | .131 | .291 | .171 | .010 | .202 | -.232 | .478 | .250 | .208 | 1 | .699 | .456 | .532 |
| Virtual reality-based simul. | .157 | .360 | .032 | -.140 | .006 | -.230 | .464 | .224 | .231 | .699 | 1 | .506 | .545 |
| Observ. simul. techniques | .312 | .437 | .220 | .207 | .218 | .161 | .566 | .441 | .365 | .456 | .506 | 1 | .696 |
| Time sequenced simul. | .382 | .444 | .041 | -.024 | .259 | -.142 | .441 | .477 | .338 | .532 | .545 | .696 | 1 |
|  | |  |  |  |  |  |  |  |  |  |  |  |  |
| Mean Spearman's rho | .255 |  |  |  |  |  |  |  |  |  |  |  |  |

**Table N4. Correlation matrix for the 13 items in the (Q27_2) assigned importance scale, showing Spearman’s *ρ* as the correlation coefficient. The mean value,** ***ρ* = 0.26, indicates a modest degree of overlap between the scale items.**


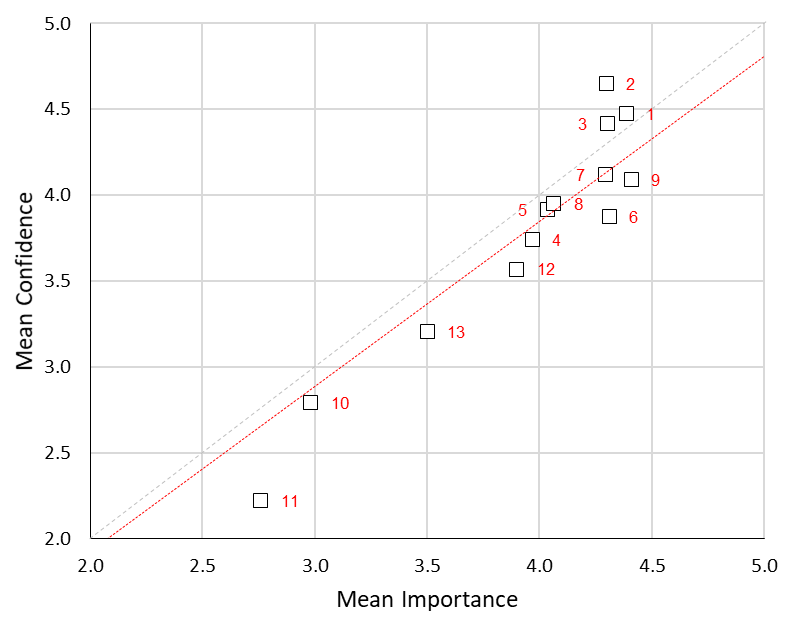


**Figure N3.** **Scatterplot of Mean Confidence *vs*. Mean Importance for the 13 items in the Q27 scale. The red dotted line is the OLS line of best fit to the data. The grey dotted line is the 1:1 line. The plot labels 1 – 13 correspond to Q27_*_1 to Q27_*_13.**

| **Test of Normality** | | | |  |
| --- | --- | --- | --- | --- |
|  | Shapiro-Wilk | | | |
|  | Statistic | df | Sig. | |
| Your confidence in – Overall (Q27_1) | .977 | 69 | .247 | |
| The importance of – Overall (Q27_2) | .971 | 69 | .110 | |

**Table N5. Results of the test for normality on the aggregated mean responses to Q27_1_1-13 and Q27_2_1-13, respectively.** **For neither Q27_1 nor Q27_2 does the data depart from normality to a significant degree (at the *p* < 0.05 level). This indicates that we could use the simple Paired-Sample t-test for the difference between the distributions. However, for consistency, we will use the nonparametric Wilcoxon Signed Rank Test in all cases.**

| **Wilcoxon Signed Rank Test** | | |
| --- | --- | --- |
|  | [The importance of – Overall (Q27_1)] –  [Your confidence in – Overall (Q27_2)] |  |
| Z | -2.154***** |  |
| Asymp. Sig. (2-tailed) | .031 |  |

**Table N6. Results of the Wilcoxon Signed Rank Test on the matched differences between aggregated mean responses to Q27_1 and Q27_2, respectively. The responses are different to a significant degree, at the *p* < 0.05 level.**

| **Reliability Statistics** | | |
| --- | --- | --- |
| N of Items | Cronbach's Alpha | |
|  | Confidence | Importance |
| 13 | .849 | .798 |
|  |  |  |
| **Item-Total Statistics** |  |  |
|  | Cronbach's Alpha if Item Deleted | Cronbach's Alpha if Item Deleted |
| Part task trainer | .837 | .787 |
| Low technology manikins | .840 | .772 |
| Medium technology manikins | .840 | .797 |
| High technology manikins | .844 | .802 |
| Your confidence in - Hybrid simulation | .837 | .790 |
| Immersive full-scale simulation | .841 | .812 |
| Conducting pause and discuss | .837 | .771 |
| In-situ simulation | .842 | .789 |
| Simulated/standardised patient simulations | .839 | .793 |
| Computer or web-based simulation | .848 | .776 |
| Virtual reality-based simulation | .843 | .776 |
| Observational simulation techniques | .821 | .761 |
| Time sequenced simulations | .821 | .761 |

**Table N7. Results of running a Reliability Analysis on the 13-item Q27 scale for both Confidence and Importance, using Cronbach’s Alpha.**

**Section P – Question 28
*[Answered only by those who self-identified as ‘Simulation Users’ in Q12, n ≈ 70]***

| **Q28_1 - Rate your confidence in doing these work items:** | | | | | | | | | | | | | | | | |
| --- | --- | --- | --- | --- | --- | --- | --- | --- | --- | --- | --- | --- | --- | --- | --- | --- |
| Your Confidence in | Virtual reality  based simulation | Computer or web- based simulation | Simulated patient  training program | Simulated patient  recruitment | Moulage techniques | Sustainability of  simulation programs | Conducting learning  needs analysis | Designing scenarios to  address quality and risk data | Summative  assessment | Interprofessional  education | Constructively aligning  scenarios with ILOs | Planning and conducting  evaluation activities | Integrating simulation  activities into curricula /programs | Formative  assessment | Teaching non-technical  skills | Team based  training |
| Question Label | Q28_1_10 | Q28_1_9 | Q28_1_7 | Q28_1_6 | Q28_1_8 | Q28_1_11 | Q28_1_1 | Q28_1_5 | Q28_1_13 | Q28_1_15 | Q28_1_3 | Q28_1_4 | Q28_1_2 | Q28_1_12 | Q28_1_14 | Q28_1_16 |
|  | Number of Respondents | | | | | | | | | | | | | | | |
| Extrem. conf. | 3 | 3 | 10 | 11 | 6 | 11 | 19 | 16 | 19 | 24 | 23 | 22 | 26 | 22 | 28 | 26 |
| Most conf. | 8 | 11 | 9 | 14 | 23 | 26 | 17 | 22 | 21 | 19 | 21 | 24 | 21 | 24 | 22 | 25 |
| Confident | 10 | 15 | 14 | 12 | 17 | 13 | 20 | 16 | 14 | 14 | 12 | 16 | 11 | 14 | 11 | 11 |
| Some. conf. | 14 | 18 | 16 | 16 | 14 | 13 | 9 | 9 | 7 | 10 | 7 | 4 | 8 | 4 | 5 | 5 |
| Not conf. | 17 | 10 | 9 | 6 | 6 | 5 | 3 | 3 | 3 | 1 | 2 | 2 | 2 | 1 | 2 | 1 |
| Total n | 52 | 57 | 58 | 59 | 66 | 68 | 68 | 66 | 64 | 68 | 65 | 68 | 68 | 65 | 68 | 68 |
|  | Percentage of Respondents | | | | | | | | | | | | | | | |
| Extrem. conf. | 5.8 | 5.3 | 17.2 | 18.6 | 9.1 | 16.2 | 27.9 | 24.2 | 29.7 | 35.3 | 35.4 | 32.4 | 38.2 | 33.8 | 41.2 | 38.2 |
| Most conf. | 15.4 | 19.3 | 15.5 | 23.7 | 34.8 | 38.2 | 25.0 | 33.3 | 32.8 | 27.9 | 32.3 | 35.3 | 30.9 | 36.9 | 32.4 | 36.8 |
| Confident | 19.2 | 26.3 | 24.1 | 20.3 | 25.8 | 19.1 | 29.4 | 24.2 | 21.9 | 20.6 | 18.5 | 23.5 | 16.2 | 21.5 | 16.2 | 16.2 |
| Some. conf. | 26.9 | 31.6 | 27.6 | 27.1 | 21.2 | 19.1 | 13.2 | 13.6 | 10.9 | 14.7 | 10.8 | 5.9 | 11.8 | 6.2 | 7.4 | 7.4 |
| Not conf. | 32.7 | 17.5 | 15.5 | 10.2 | 9.1 | 7.4 | 4.4 | 4.5 | 4.7 | 1.5 | 3.1 | 2.9 | 2.9 | 1.5 | 2.9 | 1.5 |
| Total % | 100 | 100 | 100 | 100 | 100 | 100 | 100 | 100 | 100 | 100 | 100 | 100 | 100 | 100 | 100 | 100 |

**Table P1. Number and percentage of respondents resolved according to their assigned confidence in performing certain work items. (Likert scale 1 – 5: Not confident – Extremely confident).**

| **Q28_2 – Rate the importance of the following items to your work role:** | | | | | | | | | | | | | | | | |  |
| --- | --- | --- | --- | --- | --- | --- | --- | --- | --- | --- | --- | --- | --- | --- | --- | --- | --- |
| The Importance of | Sustainability of  simulation programs | Virtual reality  based simulation | Moulage techniques | Simulated patient  training program | Computer or web- based simulation | Summative  assessment | Conducting learning  needs analysis | Simulated patient  recruitment | Integrating simulation  activities into curricula /programs | Planning and conducting  evaluation activities | Interprofessional  education | Designing scenarios to  address quality and risk data | Constructively aligning  scenarios with ILOs | Formative  assessment | Team based  training | Teaching non-technical  skills | |
| Question Label | Q28_2_11 | Q28_2_10 | Q28_2_8 | Q28_2_7 | Q28_2_9 | Q28_2_13 | Q28_2_1 | Q28_2_6 | Q28_2_2 | Q28_2_4 | Q28_2_15 | Q28_2_5 | Q28_2_3 | Q28_2_12 | Q28_2_16 | Q28_2_14 | |
|  | Number of Respondents | | | | | | | | | | | | | | | | |
| Extrem. import. | 7 | 10 | 18 | 16 | 15 | 30 | 34 | 39 | 42 | 45 | 49 | 48 | 50 | 48 | 50 | 47 | |
| Mod. Import. | 8 | 8 | 18 | 20 | 26 | 18 | 21 | 15 | 16 | 13 | 8 | 12 | 9 | 13 | 11 | 15 | |
| Some. Import. | 13 | 17 | 7 | 14 | 13 | 8 | 6 | 7 | 7 | 4 | 9 | 6 | 7 | 5 | 2 | 3 | |
| Least import. | 15 | 14 | 12 | 6 | 9 | 4 | 2 | 5 | 3 | 2 | 2 | 2 | 2 | 2 | 3 | 2 | |
| Not import. | 10 | 9 | 5 | 5 | 3 | 3 | 1 | 0 | 0 | 1 | 0 | 0 | 0 | 0 | 1 | 0 | |
| Total n | 53 | 58 | 60 | 61 | 66 | 63 | 64 | 66 | 68 | 65 | 68 | 68 | 68 | 68 | 67 | 67 | |
|  | Percentage of Respondents | | | | | | | | | | | | | | | | |
| Extrem. import. | 13.2 | 17.2 | 30.0 | 26.2 | 22.7 | 47.6 | 53.1 | 59.1 | 61.8 | 69.2 | 72.1 | 70.6 | 73.5 | 70.6 | 74.6 | 70.1 | |
| Mod. Import. | 15.1 | 13.8 | 30.0 | 32.8 | 39.4 | 28.6 | 32.8 | 22.7 | 23.5 | 20.0 | 11.8 | 17.6 | 13.2 | 19.1 | 16.4 | 22.4 | |
| Some. Import. | 24.5 | 29.3 | 11.7 | 23.0 | 19.7 | 12.7 | 9.4 | 10.6 | 10.3 | 6.2 | 13.2 | 8.8 | 10.3 | 7.4 | 3.0 | 4.5 | |
| Least import. | 28.3 | 24.1 | 20.0 | 9.8 | 13.6 | 6.3 | 3.1 | 7.6 | 4.4 | 3.1 | 2.9 | 2.9 | 2.9 | 2.9 | 4.5 | 3.0 | |
| Not import. | 18.9 | 15.5 | 8.3 | 8.2 | 4.5 | 4.8 | 1.6 | 0.0 | 0.0 | 1.5 | 0.0 | 0.0 | 0.0 | 0.0 | 1.5 | 0.0 | |
| Total % | 100 | 100 | 100 | 100 | 100 | 100 | 100 | 100 | 100 | 100 | 100 | 100 | 100 | 100 | 100 | 100 | |

**Table P2. Number and percentage of respondents resolved according to their assigned importance to certain items in their work role. (Likert scale 1 – 5: Not important – Extremely important).**

| Your Confidence in | Conducting learning needs analysis | Integrating simulation activities into curricula/programs | Constructively aligning scenarios with ILOs | Planning and conducting evaluation activities | Designing scenarios to address quality and risk data | Simulated patient recruitment | Simulated patient training program | Moulage techniques | Computer or web-based simulation | Virtual reality-based simulation | Sustainability of simulation programs | Formative assessment | Summative assessment | Teaching non-technical skills | Interprofessional education | Team based training |
| --- | --- | --- | --- | --- | --- | --- | --- | --- | --- | --- | --- | --- | --- | --- | --- | --- |
| Conducting learning needs analysis | 1 | .656 | .566 | .760 | .579 | .414 | .476 | .284 | .453 | .457 | .577 | .490 | .481 | .602 | .534 | .483 |
| Integrating sim. activities into curricula | .656 | 1 | .674 | .753 | .621 | .447 | .384 | .379 | .366 | .333 | .650 | .692 | .644 | .764 | .592 | .584 |
| Constructively aligning scenarios with ILOs | .566 | .674 | 1 | .740 | .761 | .484 | .478 | .195 | .337 | .345 | .484 | .617 | .683 | .524 | .476 | .476 |
| Planning/conduct evaluation activities | .760 | .753 | .740 | 1 | .789 | .455 | .432 | .339 | .415 | .448 | .598 | .617 | .609 | .664 | .592 | .584 |
| Design scenarios to address quality/risk data | .579 | .621 | .761 | .789 | 1 | .378 | .310 | .380 | .358 | .378 | .544 | .740 | .701 | .644 | .701 | .682 |
| Simulated patient recruitment | .414 | .447 | .484 | .455 | .378 | 1 | .844 | .253 | .488 | .496 | .509 | .323 | .331 | .337 | .254 | .169 |
| Simulated patient training program | .476 | .384 | .478 | .432 | .310 | .844 | 1 | .229 | .572 | .547 | .504 | .281 | .335 | .287 | .182 | .113 |
| Moulage techniques | .284 | .379 | .195 | .339 | .380 | .253 | .229 | 1 | .356 | .264 | .413 | .327 | .307 | .450 | .386 | .462 |
| Computer or web-based simulation | .453 | .366 | .337 | .415 | .358 | .488 | .572 | .356 | 1 | .794 | .582 | .346 | .498 | .390 | .379 | .292 |
| Virtual reality-based simulation | .457 | .333 | .345 | .448 | .378 | .496 | .547 | .264 | .794 | 1 | .535 | .299 | .402 | .351 | .376 | .332 |
| Sustainability of sim. programs | .577 | .650 | .484 | .598 | .544 | .509 | .504 | .413 | .582 | .535 | 1 | .482 | .629 | .496 | .494 | .474 |
| Formative assessment | .490 | .692 | .617 | .617 | .740 | .323 | .281 | .327 | .346 | .299 | .482 | 1 | .833 | .730 | .648 | .670 |
| Summative assessment | .481 | .644 | .683 | .609 | .701 | .331 | .335 | .307 | .498 | .402 | .629 | .833 | 1 | .662 | .585 | .577 |
| Teaching non-technical skills | .602 | .764 | .524 | .664 | .644 | .337 | .287 | .450 | .390 | .351 | .496 | .730 | .662 | 1 | .665 | .768 |
| Interprofessional education | .534 | .592 | .476 | .592 | .701 | .254 | .182 | .386 | .379 | .376 | .494 | .648 | .585 | .665 | 1 | .838 |
| Team based training | .483 | .584 | .476 | .584 | .682 | .169 | .113 | .462 | .292 | .332 | .474 | .670 | .577 | .768 | .838 | 1 |
|  |  |  |  |  |  |  |  |  |  |  |  |  |  |  |  |  |
| Mean Spearman's rho | .498 |  |  |  |  |  |  |  |  |  |  |  |  |  |  |  |

**Table P3. Correlation matrix for the 16 items in the (Q28_1) assigned confidence scale, showing Spearman’s *ρ* as the correlation coefficient.** **The mean value, *ρ* = 0.50, indicates a moderate degree of overlap between the scale items.**

| The Importance of | Conducting learning needs analysis | Integrating simulation activities into curricula/programs | Constructively aligning scenarios with ILOs | Planning and conducting evaluation activities | Designing scenarios to address quality and risk data | Simulated patient recruitment | Simulated patient training program | Moulage techniques | Computer or web-based simulation | Virtual reality-based simulation | Sustainability of simulation programs | Formative assessment | Summative assessment | Teaching non-technical skills | Interprofessional education | Team based training |
| --- | --- | --- | --- | --- | --- | --- | --- | --- | --- | --- | --- | --- | --- | --- | --- | --- |
| Conducting learning needs analysis | 1 | .537 | .409 | .573 | .536 | .251 | .333 | .061 | .148 | .371 | .389 | .427 | .331 | .324 | .425 | .411 |
| Integrating sim. activities into curricula | .537 | 1 | .698 | .686 | .495 | .286 | .200 | .125 | .170 | .269 | .396 | .306 | .201 | .487 | .385 | .291 |
| Constructively aligning scenarios with ILOs | .409 | .698 | 1 | .742 | .784 | .413 | .305 | .056 | .288 | .292 | .362 | .517 | .449 | .478 | .343 | .244 |
| Planning/conduct evaluation activities | .573 | .686 | .742 | 1 | .707 | .413 | .343 | .073 | .210 | .312 | .512 | .440 | .365 | .376 | .437 | .366 |
| Design scenarios to address quality/risk data | .536 | .495 | .784 | .707 | 1 | .358 | .294 | .129 | .202 | .263 | .358 | .609 | .421 | .383 | .421 | .494 |
| Simulated patient recruitment | .251 | .286 | .413 | .413 | .358 | 1 | .856 | .315 | .546 | .502 | .444 | .244 | .272 | .137 | .308 | .225 |
| Simulated patient training program | .333 | .200 | .305 | .343 | .294 | .856 | 1 | .294 | .484 | .510 | .399 | .274 | .249 | .049 | .257 | .193 |
| Moulage techniques | .061 | .125 | .056 | .073 | .129 | .315 | .294 | 1 | .459 | .256 | .270 | .313 | .262 | .150 | .410 | .267 |
| Computer or web-based simulation | .148 | .170 | .288 | .210 | .202 | .546 | .484 | .459 | 1 | .759 | .218 | .227 | .649 | .160 | .246 | .048 |
| Virtual reality-based simulation | .371 | .269 | .292 | .312 | .263 | .502 | .510 | .256 | .759 | 1 | .229 | .356 | .532 | .254 | .300 | .134 |
| Sustainability of sim. programs | .389 | .396 | .362 | .512 | .358 | .444 | .399 | .270 | .218 | .229 | 1 | .303 | .292 | .458 | .521 | .498 |
| Formative assessment | .427 | .306 | .517 | .440 | .609 | .244 | .274 | .313 | .227 | .356 | .303 | 1 | .513 | .453 | .408 | .367 |
| Summative assessment | .331 | .201 | .449 | .365 | .421 | .272 | .249 | .262 | .649 | .532 | .292 | .513 | 1 | .328 | .247 | .126 |
| Teaching non-technical skills | .324 | .487 | .478 | .376 | .383 | .137 | .049 | .150 | .160 | .254 | .458 | .453 | .328 | 1 | .462 | .519 |
| Interprofessional education | .425 | .385 | .343 | .437 | .421 | .308 | .257 | .410 | .246 | .300 | .521 | .408 | .247 | .462 | 1 | .657 |
| Team based training | .411 | .291 | .244 | .366 | .494 | .225 | .193 | .267 | .048 | .134 | .498 | .367 | .126 | .519 | .657 | 1 |
|  |  |  |  |  |  |  |  |  |  |  |  |  |  |  |  |  |
| Mean Spearman's rho | .362 |  |  |  |  |  |  |  |  |  |  |  |  |  |  |  |

**Table P4. Correlation matrix for the 16 items in the (Q28_2) assigned importance scale, showing** **Spearman’s *ρ* as the correlation coefficient. The mean value,** ***ρ* = 0.36, indicates a moderate degree of overlap between the scale items.**


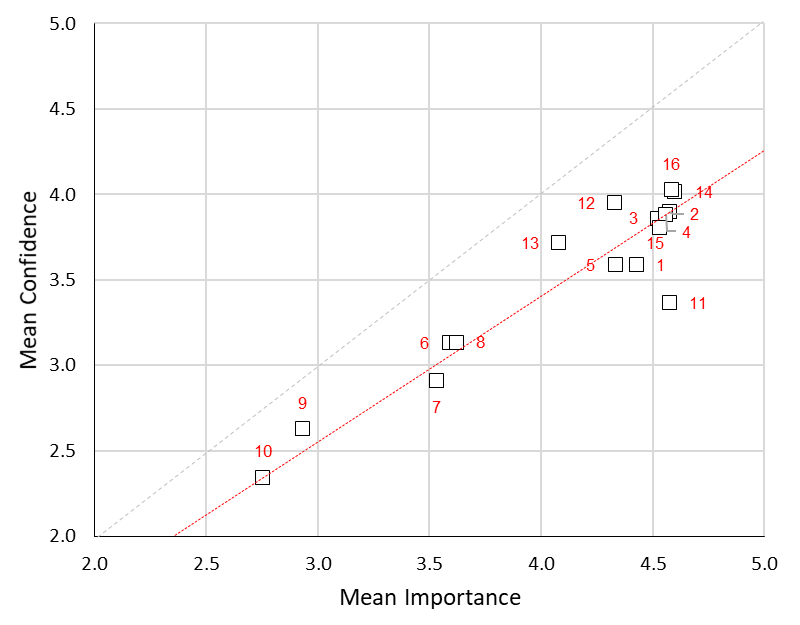


**Figure P3.** **Scatterplot of Mean Confidence *vs*. Mean Importance for the 16 items in the Q28 scale. The red dotted line is the OLS line of best fit to the data. The grey dotted line is the 1:1 line. The plot labels 1 – 16 correspond to Q28_*_1 to Q28_*_16.**

| **Test of Normality** | | | |  |
| --- | --- | --- | --- | --- |
|  | Shapiro-Wilk | | | |
|  | Statistic | df | Sig. | |
| Your confidence in – Overall (Q28_1) | .978 | 70 | .254 | |
| The importance of – Overall (Q28_2) | .937**** | 70 | .002 | |

**Table P5. Results of the test for normality on the aggregated mean responses to Q28_1_1-16 and Q28_2_1-16, respectively. For Q22_2,** **the data departs significantly (*p* < 0.01) from normality. This indicates that a nonparametric test for differences between the matched mean responses should be used (e.g. the Wilcoxon Signed Rank Test).**

| **Wilcoxon Signed Rank Test** | |  |
| --- | --- | --- |
|  | [The importance of – Overall (Q28_2)] –  [Your confidence in – Overall (Q28_1)] | |
| Z | -4.872****** | |
| Asymp. Sig. (2-tailed) | < .001 | |

**Table P6. Results of** **the Wilcoxon Signed Rank Test on the matched differences between aggregated mean responses to Q28_1 and Q28_2, respectively. The responses are different to a significant degree, at the *p* < 0.01 level.**

| **Reliability Statistics** | | |
| --- | --- | --- |
| N of Items | Cronbach's Alpha | |
|  | Confidence | Importance |
| 16 | .940 | .903 |
|  |  |  |
| **Item-Total Statistics** |  |  |
| Item | Cronbach's Alpha if Item Deleted | Cronbach's Alpha if Item Deleted |
| Conducting learning needs analysis | .935 | .895 |
| Integrating simulation activities into curricula/programs | .932 | .896 |
| Constructively aligning scenarios with ILOs | .934 | .894 |
| Planning and conducting evaluation activities | .933 | .893 |
| Designing scenarios to address quality and risk data | .934 | .892 |
| Simulated patient recruitment | .939 | .897 |
| Simulated patient training program | .939 | .896 |
| Moulage techniques | .941 | .908 |
| Computer or web-based simulation | .938 | .899 |
| Virtual reality-based simulation | .938 | .899 |
| Sustainability of simulation programs | .935 | .898 |
| Formative assessment | .936 | .897 |
| Summative assessment | .935 | .899 |
| Teaching non-technical skills | .934 | .900 |
| Interprofessional education | .936 | .896 |
| Team based training | .935 | .899 |

**Table P7. Results of running a Reliability Analysis on the 10-item Q20 scale for both Confidence and Importance, using Cronbach’s Alpha.**

***[Professional Development Section***

| **Q37_1 – Rate your confidence in research related to simulation in your profession/work area:** | | | | | | | | | |  |  |
| --- | --- | --- | --- | --- | --- | --- | --- | --- | --- | --- | --- |
| Your confidence in | Developing budgets  for grant funding | Locating sources of  funding | Applying for grant  funding | Developing research  protocols | Writing for publication | Research ethics | Developing and using  tools and instruments  to measure outcome | Writing and reporting  project outcomes | Writing a conference  abstract | | Developing a conference  presentation |
| Question Label | Q37_1_5 | Q37_1_3 | Q37_1_4 | Q37_1_1 | Q37_1_7 | Q37_1_10 | Q37_1_2 | Q37_1_6 | Q37_1_8 | | Q37_1_9 |
|  | Number of Respondents | | | | | | | | | | |
| Extremely confident | 3 | 6 | 5 | 5 | 7 | 10 | 5 | 9 | 14 | | 14 |
| Mostly confident | 7 | 4 | 7 | 11 | 12 | 12 | 14 | 10 | 11 | | 15 |
| Confident | 11 | 14 | 10 | 7 | 8 | 12 | 20 | 20 | 14 | | 17 |
| Somewhat confident | 20 | 16 | 20 | 29 | 28 | 23 | 27 | 26 | 24 | | 21 |
| Not confident | 46 | 46 | 45 | 35 | 34 | 31 | 23 | 23 | 25 | | 22 |
| Total n | 87 | 86 | 87 | 88 | 89 | 88 | 89 | 88 | 88 | | 89 |
|  | Percentage of Respondents | | | | | | | | | | |
| Extremely confident | 3.4 | 7.0 | 5.7 | 5.7 | 7.9 | 11.4 | 5.6 | 10.2 | 15.9 | | 15.7 |
| Mostly confident | 8.0 | 4.7 | 8.0 | 12.5 | 13.5 | 13.6 | 15.7 | 11.4 | 12.5 | | 16.9 |
| Confident | 12.6 | 16.3 | 11.5 | 8.0 | 9.0 | 13.6 | 22.5 | 22.7 | 15.9 | | 19.1 |
| Somewhat confident | 23.0 | 18.6 | 23.0 | 33.0 | 31.5 | 26.1 | 30.3 | 29.5 | 27.3 | | 23.6 |
| Not confident | 52.9 | 53.5 | 51.7 | 39.8 | 38.2 | 35.2 | 25.8 | 26.1 | 28.4 | | 24.7 |
| Total % | 100 | 100 | 100 | 100 | 100 | 100 | 100 | 100 | 100 | | 100 |

**Table V1. Number and percentage of respondents resolved according to their assigned confidence in research related to simulation in their profession/work area. (Likert scale 1 – 5: Not confident – Extremely confident).**

| **Q37_2 - Rate the importance of research related to simulation in your profession/work area:** | | | | | | | | |  |  |  |
| --- | --- | --- | --- | --- | --- | --- | --- | --- | --- | --- | --- |
| The importance of | Developing research  protocols | Writing a conference  abstract | Developing a conference  presentation | Developing budgets  for grant funding | Research ethics | Applying for grant  funding | Locating sources of  funding | Writing for publication | | Developing and using  tools and instruments  to measure outcome | Writing and reporting  project outcomes |
| Question Label | Q37_2_1 | Q37_2_8 | Q37_2_9 | Q37_2_5 | Q37_2_10 | Q37_2_4 | Q37_2_3 | Q37_2_7 | | Q37_2_2 | Q37_2_6 |
|  | Number of Respondents | | | | | | | | | | |
| Extremely important | 20 | 26 | 24 | 32 | 29 | 35 | 34 | 31 | | 33 | 36 |
| Moderately important | 30 | 35 | 38 | 25 | 32 | 22 | 25 | 30 | | 34 | 35 |
| Somewhat important | 25 | 19 | 19 | 17 | 17 | 20 | 16 | 19 | | 17 | 11 |
| Least important | 10 | 6 | 5 | 11 | 8 | 9 | 11 | 5 | | 2 | 7 |
| Not at all important | 3 | 4 | 4 | 3 | 3 | 3 | 2 | 4 | | 3 | 1 |
| Total n | 88 | 90 | 90 | 88 | 89 | 89 | 88 | 89 | | 89 | 90 |
|  | Percentage of Respondents | | | | | | | | | | |
| Extremely important | 22.7 | 28.9 | 26.7 | 36.4 | 32.6 | 39.3 | 38.6 | 34.8 | | 37.1 | 40.0 |
| Moderately important | 34.1 | 38.9 | 42.2 | 28.4 | 36.0 | 24.7 | 28.4 | 33.7 | | 38.2 | 38.9 |
| Somewhat important | 28.4 | 21.1 | 21.1 | 19.3 | 19.1 | 22.5 | 18.2 | 21.3 | | 19.1 | 12.2 |
| Least important | 11.4 | 6.7 | 5.6 | 12.5 | 9.0 | 10.1 | 12.5 | 5.6 | | 2.2 | 7.8 |
| Not at all important | 3.4 | 4.4 | 4.4 | 3.4 | 3.4 | 3.4 | 2.3 | 4.5 | | 3.4 | 1.1 |
| Total % | 100 | 100 | 100 | 100 | 100 | 100 | 100 | 100 | | 100 | 100 |

**Table V2. Number and percentage of respondents resolved according to their assigned importance to research related to simulation in their profession/work area. (Likert scale 1 – 5: Not at all important – Extremely important).**

| Your Confidence in | Developing research protocols | Developing and using tools and instruments to measure outcome | Locating sources of funding | Applying for grant funding | Developing budgets for grant funding | Writing and reporting project outcomes | Writing for publication | Writing a conference abstract | Developing a conference presentation | Research ethics |
| --- | --- | --- | --- | --- | --- | --- | --- | --- | --- | --- |
| Developing research protocols | 1 | .766 | .675 | .667 | .635 | .583 | .635 | .595 | .580 | .641 |
| Developing and using tools and instruments to measure outcome | .766 | 1 | .690 | .636 | .613 | .544 | .483 | .384 | .419 | .497 |
| Locating sources of funding | .675 | .690 | 1 | .878 | .791 | .590 | .571 | .461 | .448 | .563 |
| Applying for grant funding | .667 | .636 | .878 | 1 | .898 | .652 | .613 | .520 | .505 | .589 |
| Your confidence in - Developing budgets for grant funding | .635 | .613 | .791 | .898 | 1 | .707 | .532 | .475 | .491 | .560 |
| Writing and reporting project outcomes | .583 | .544 | .590 | .652 | .707 | 1 | .645 | .612 | .585 | .662 |
| Writing for publication | .635 | .483 | .571 | .613 | .532 | .645 | 1 | .874 | .797 | .847 |
| Writing a conference abstract | .595 | .384 | .461 | .520 | .475 | .612 | .874 | 1 | .934 | .825 |
| Developing a conference presentation | .580 | .419 | .448 | .505 | .491 | .585 | .797 | .934 | 1 | .783 |
| Research ethics | .641 | .497 | .563 | .589 | .560 | .662 | .847 | .825 | .783 | 1 |
|  |  |  |  |  |  |  |  |  |  |  |
| Mean Spearman's rho | 0.632 |  |  |  |  |  |  |  |  |  |

**Table V3. Correlation matrix for the ten items in the (Q37_1) assigned confidence scale, showing Spearman’s *ρ* as the correlation coefficient. The mean value,** ***ρ* = 0.63, indicates a moderately high degree of overlap between the scale items.**

| The Importance of | Developing research protocols | Developing and using tools and instruments to measure outcome | Locating sources of funding | Applying for grant funding | Developing budgets for grant funding | Writing and reporting project outcomes | Writing for publication | Writing a conference abstract | Developing a conference presentation | Research ethics |
| --- | --- | --- | --- | --- | --- | --- | --- | --- | --- | --- |
| Developing research protocols | 1 | .647 | .621 | .610 | .630 | .640 | .608 | .559 | .522 | .711 |
| Developing and using tools and instruments to measure outcome | .647 | 1 | .561 | .546 | .591 | .680 | .635 | .516 | .499 | .664 |
| Locating sources of funding | .621 | .561 | 1 | .873 | .909 | .730 | .615 | .508 | .496 | .567 |
| Applying for grant funding | .610 | .546 | .873 | 1 | .907 | .776 | .626 | .473 | .475 | .581 |
| Your confidence in - Developing budgets for grant funding | .630 | .591 | .909 | .907 | 1 | .767 | .581 | .508 | .496 | .554 |
| Writing and reporting project outcomes | .640 | .680 | .730 | .776 | .767 | 1 | .786 | .680 | .634 | .745 |
| Writing for publication | .608 | .635 | .615 | .626 | .581 | .786 | 1 | .824 | .796 | .848 |
| Writing a conference abstract | .559 | .516 | .508 | .473 | .508 | .680 | .824 | 1 | .944 | .744 |
| Developing a conference presentation | .522 | .499 | .496 | .475 | .496 | .634 | .796 | .944 | 1 | .729 |
| Research ethics | .711 | .664 | .567 | .581 | .554 | .745 | .848 | .744 | .729 | 1 |
|  |  |  |  |  |  |  |  |  |  |  |
| Mean Spearman's rho | 0.654 |  |  |  |  |  |  |  |  |  |

**Table V4. Correlation matrix for the ten items in the (Q37_2) assigned importance scale, showing Spearman’s *ρ* as the correlation coefficient. The mean value,** ***ρ* = 0.65, indicates a moderately high degree of overlap between the scale items.**


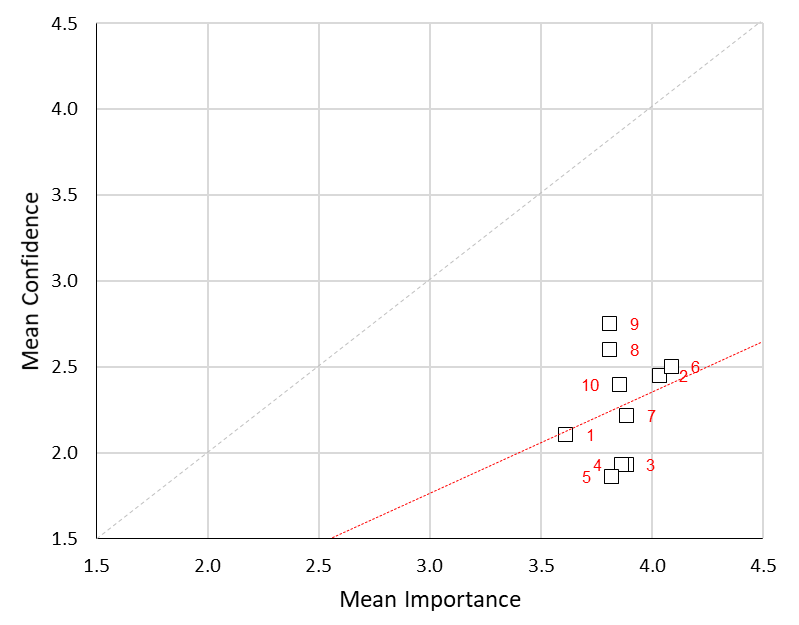


**Figure V3.** **Scatterplot of Mean Confidence *vs*. Mean Importance for the ten items in the Q37 scale. The red dotted line is the OLS line of best fit to the data. The grey dotted line is the 1:1 line. The plot labels 1 – 10 correspond to Q37_*_1 to Q37_*_10.**

| **Test for Normality** | | | |  |
| --- | --- | --- | --- | --- |
|  | Shapiro-Wilk | | | |
|  | Statistic | df | Sig. | |
| Your confidence in – Overall (Q37_1) | .889****** | 90 | < .001 | |
| The importance of – Overall (Q37_2) | .939****** | 90 | < .001 | |

**Table V5. Results of the test for normality on the aggregated mean responses to Q37_1_1-10 and Q37_2_1-10, respectively. For both Q37_1 and Q37_2,** **the data departs significantly (*p* < 0.01) from normality. This indicates that a nonparametric test for differences between the matched mean responses should be used (e.g. the Wilcoxon Signed Rank Test).**

| **Wilcoxon Signed Rank Test** | | |
| --- | --- | --- |
|  | [The importance of – Overall (Q37_1)] –  [Your confidence in – Overall (Q37_1)] |  |
| Z | -7.675****** |  |
| Asymp. Sig. (2-tailed) | < .001 |  |

**Table V6. Results of** **the Wilcoxon Signed Rank Test on the matched differences between aggregated mean responses to Q37_1 and Q37_2, respectively. The responses are different to a significant degree, at the *p* < 0.01 level.**

| **Reliability Statistics** | | |
| --- | --- | --- |
| N of Items | Cronbach's Alpha | |
|  | Confidence | Importance |
| 10 | .957 | .940 |
|  |  |  |
| **Item-Total Statistics** | | |
| Item | Cronbach's Alpha if Item Deleted | Cronbach's Alpha if Item Deleted |
| Developing research protocols | .951 | .935 |
| Developing and using tools and instruments to measure outcome | .956 | .937 |
| Locating sources of funding | .954 | .934 |
| Applying for grant funding | .952 | .934 |
| Developing budgets for grant funding | .953 | .934 |
| Writing and reporting project outcomes | .954 | .930 |
| Writing for publication | .951 | .932 |
| Writing a conference abstract | .953 | .935 |
| Developing a conference presentation | .954 | .937 |
| Research ethics | .951 | .931 |

**Table V7. Results of running a Reliability Analysis on the 10-item Q37 scale for both Confidence and Importance, using Cronbach’s Alpha.**
